# Whole-Genome Resequencing Reveals Genomic Signatures Underlying High-Altitude Adaptation in Ethiopian Indigenous Cattle

**DOI:** 10.3390/biology15201822

**Published:** 2026-10-12

**Authors:** Ruoshan Ma, Dongfang Li, Ying Lu, Bo Wang, Weidong Deng, Zhendong Gao

**Affiliations:** Yunnan Provincial Key Laboratory of Animal Science and Feed, Faculty of Animal Science and Technology, Yunnan Agricultural University, Kunming 650201, China; maruoshan_2000@163.com (R.M.); dfli0927@163.com (D.L.); yinglu_1998@163.com (Y.L.); wangbo@ynau.edu.cn (B.W.)

**Keywords:** whole-genome resequencing, high-altitude adaptation, Ethiopian cattle, single-cell virtual knockout

## Abstract

High-altitude environments impose severe physiological challenges on mammals, yet the genetic basis of adaptation in Ethiopian indigenous cattle remains poorly understood. Here, we analyzed whole-genome resequencing data from 70 Ethiopian indigenous cattle representing seven breeds (Abigar, Barka, Boran, Felata, Fogera, Gojjam-Highland, and Horro) distributed across elevations ranging from 523 to 3410 m, obtained from the NCBI Sequence Read Archive. After alignment to the ARS-UCD2.0 reference genome, we identified 49,196,624 high-confidence SNPs and performed population genomic analyses (PCA, phylogenetic reconstruction, and ADMIXTURE), along with genome-wide selection scans using FST and nucleotide diversity (θπ), and in silico virtual knockout of candidate genes in bovine lung single-cell transcriptomics. Population structure analyses revealed marked genetic differentiation between low- and mid-to-high-altitude populations, with substantial genetic connectivity and shared ancestry among high-altitude groups. We identified 398 candidate selective regions showing concordant FST and θπ signals, encompassing 1816 candidate genes, significantly enriched in hypoxia-related and vascular remodeling pathways (phospholipase D, Ras, ErbB, Wnt, Hedgehog). Two hub candidate genes, *PIK3CA* and *RHOA*, were prioritized; virtual perturbation of *PIK3CA* altered extracellular matrix, focal adhesion, and integrin networks, whereas *RHOA* perturbation activated immune and inflammatory programs (NF-κB, phagocytosis, chemokine pathways) with upregulation of *TYROBP*, *IL1B*, *TLR4*, and *NLRP3*. Our findings indicate that Ethiopian cattle have evolved distinct genomic signatures associated with adaptation to chronic high-altitude environments, in which *PIK3CA* and *RHOA* may represent candidate regulators associated with extracellular matrix organization and immune-related transcriptional regulation, respectively, thereby providing new insights into mammalian adaptation to extreme environments.

## Simple Summary

Living at high altitudes is challenging for animals because the air contains less oxygen. Cattle in the Ethiopian Highlands have lived there for a long time and may have developed special genetic traits to cope with this harsh environment. In this study, we compared the complete genetic codes of 70 Ethiopian cattle living at different heights above sea level, from lowlands to very high mountains. We found that cattle from high-altitude areas have clear genetic differences from those in lowland areas, suggesting that they have adapted over time. We identified several genes that are likely responsible for this adaptation, especially two key genes that help maintain healthy lung structure and control the body’s immune responses. These genetic changes may help the cattle breathe more efficiently and stay healthy under low-oxygen conditions. Understanding these natural adaptations not only helps us protect these valuable local animals but may also provide useful knowledge for improving the health of other animals and possibly even humans living in similar extreme environments.

## 1. Introduction

Modern humans dispersed out of Africa at least 60,000 years ago and progressively colonized a wide range of environments, including high-altitude regions exceeding 2500 m above sea level [1,2]. Today, the major long-term high-altitude human populations include inhabitants of the Tibetan Plateau, the Andean Highlands, and the Ethiopian Highlands [3]. Extensive studies have demonstrated that prolonged exposure to chronic hypoxia has driven the evolution of distinct physiological and genetic adaptations in these populations, with previous research largely focusing on Asian and South American highlanders and identifying key adaptive mechanisms involved in hypoxia sensing, hemoglobin function, and energy metabolism [4,5,6,7]. The expansion of human populations into high-altitude regions was accompanied by the introduction of domesticated animals into these extreme environments. Under the combined influences of domestication and natural selection, these animals have likewise been exposed to multiple environmental stressors, including chronic hypoxia, intense ultraviolet radiation, and low temperatures, resulting in the evolution of diverse adaptive phenotypes [8]. Compared with studies of human high-altitude adaptation, investigations of adaptive evolution in domesticated animals provide not only an opportunity to identify evolutionary strategies shared across mammalian species but also an important model for understanding how natural and artificial selection interact during environmental adaptation.

The Tibetan Plateau has been regarded as a natural laboratory for investigating high-altitude adaptation, and extensive studies have explored adaptive mechanisms in both domestic and wild animals from this region. Whole-genome resequencing studies have revealed that Tibetan sheep exhibit distinct population structures and high levels of genetic diversity, with their adaptation to hypoxia being closely associated with optimization of hemoglobin function and enhanced oxygen transport capacity [9,10,11,12]. In bovine species, Wu et al. [13] performed genome-wide scans on three indigenous cattle breeds and identified a set of candidate genes associated with high-altitude adaptation, including *ANGPT1*, *PPARGC1A*, and *RORA*. Their study further revealed that these breeds possess admixed genomic components derived from both indicine and taurine cattle lineages. In yak, Qiu et al. [14] used whole-genome sequencing to identify multiple hypoxia-associated genes, including *ADAM17*, *ARHGEF5*, and members of the HIF-1α signaling pathway. Subsequent studies have further uncovered the complex population history, domestication processes, and functional genes involved in growth, development, and energy metabolism in yak populations [15,16]. Moreover, structural variations and introgression from wild relatives have been proposed as important sources of genetic variation facilitating rapid adaptation of domestic animals to high-altitude environments [17,18]. Beyond the Tibetan Plateau, the Andes provide another valuable comparative system for understanding high-altitude adaptation. Livestock populations inhabiting the Andean Highlands have evolved remarkable physiological and genetic adaptations under persistent environmental challenges, including hypoxia, aridity, and large temperature fluctuations [19]. In South American camelids, including *Lama glama* and *Vicugna pacos*, complex polymorphisms and alternative splicing patterns within milk protein gene clusters suggest functional adaptations associated with survival and reproduction at high elevations [20]. Creole sheep from the Peruvian Highlands have undergone centuries of domestication and selection, resulting in regional variation in body size, body weight, and milk production capacity while maintaining overall genomic stability [21,22,23]. Using genome-wide SNP arrays, Marcuzzi et al. [24] compared highland and lowland Bolivian Creole cattle populations and identified selective signatures associated with hypoxia response (*ANXA2*, *NDUFA4L2*) and angiogenesis (*NRP1*, *NRP2*), suggesting that highland cattle have undergone complex molecular adaptations involving oxygen sensing, immune regulation, and metabolic reprogramming.

The Ethiopian Highlands, located in the Horn of Africa, represent another important high-altitude ecosystem characterized by remarkable livestock genetic diversity. Previous studies based on microsatellite markers and SNP arrays have demonstrated substantial genetic diversity and pronounced population differentiation among Ethiopian cattle populations, with clear divergence between lowland and highland populations, potentially reflecting adaptive responses to contrasting ecological conditions [25,26]. However, compared with animals from the Tibetan and Andean plateaus, the genomic basis underlying high-altitude adaptation in Ethiopian indigenous cattle remains largely unexplored. The Simien Mountains in the Ethiopian Highlands rise above 2500 m in elevation, with annual average temperatures below 10 °C and an oxygen partial pressure approximately 60% of that at sea level [27], imposing persistent selective pressures associated with hypoxia and cold stress. Although our analyses identified genomic signatures associated with high-altitude adaptation, the environmental pressures underlying these signals are likely multifactorial. Highland environments differ from lowland regions not only in oxygen availability but also in temperature, pathogen exposure, forage composition, nutritional resources, and livestock management practices. Therefore, the identified selective regions should not be interpreted as exclusively representing hypoxia-driven adaptation. In particular, cold stress represents an important environmental component of high-altitude ecosystems and may impose selective pressures partially overlapping with hypoxia responses, such as regulation of energy metabolism, vascular function, and cellular stress pathways. Archaeological and genomic evidence suggests that Ethiopian indigenous cattle were introduced into the Horn of Africa from Egypt approximately 5000–4000 years ago [28] and subsequently experienced potential genetic interactions with indigenous wild African aurochs. Their introduction from Egypt into the Horn of Africa, followed by potential genetic interactions with local African aurochs, suggests that Ethiopian highland cattle may share conserved molecular mechanisms of high-altitude adaptation with other highland species while also exhibiting adaptive features shaped by local environmental pressures.

Against this background, Ethiopian indigenous cattle have experienced a complex combination of selective pressures, including chronic hypoxia, intense ultraviolet radiation, cold stress, and high pathogen exposure, during a relatively recent but distinctive domestication and adaptation history [28]. Nevertheless, the molecular mechanisms underlying their high-altitude adaptation remain poorly characterized. Here, we leveraged publicly available whole-genome resequencing datasets generated by Ayalew et al. [29], but applied an independent analytical framework focusing on selective sweeps, functional pathway interpretation, and transcriptomic-based computational functional inference. Unlike previous studies mainly emphasizing population structure and structural variation, including copy number variation, our study aims to uncover the evolutionary genomic signatures and potential functional mechanisms underlying high-altitude adaptation in Ethiopian indigenous cattle. By integrating population genomic analyses, genome-wide selective sweep detection, functional enrichment analyses, and single-cell transcriptome based in silico virtual knockout experiments, we identified candidate genomic regions and explored their potential functional roles in adaptation to high-altitude environments. Our study provides new insights into the molecular mechanisms underlying high-altitude adaptation in African livestock and advances our understanding of convergent and lineage-specific evolutionary strategies among domesticated animals across different highland ecosystems. Furthermore, these findings provide a theoretical foundation for molecular breeding and genetic resource conservation of cattle populations inhabiting extreme environments.

## 2. Materials and Methods

### 2.1. Data Acquisition

This study utilized all 70 Ethiopian indigenous cattle individuals available in the original whole-genome resequencing datasets generated by Ayalew et al. [29]. These individuals represented seven indigenous cattle breeds, and no additional subsampling or random selection was performed. The paired-end raw sequencing data were retrieved from the NCBI Sequence Read Archive (SRA) under accession numbers SRP478348 and SRP480803. Although these sequencing datasets were previously generated and reported by Ayalew et al. [29], the present study performed an independent reanalysis using population genomic selection scans, functional enrichment analyses, and single-cell transcriptome-based computational functional inference to investigate the molecular mechanisms underlying high-altitude adaptation in Ethiopian indigenous cattle. Based on geographic distribution and ecological gradients, we selected seven representative indigenous cattle breeds, comprising a total of 70 individuals (10 individuals per breed; Supplementary Table S1). As summarized in Table 1, the sampled populations covered three major agro-ecological zones of Ethiopia: the tropical lowland region of Gambela (Abigar), the Ethiopian highland region of Amhara (Barka, Felata, Fogera, and Gojjam-Highland), and the mid-altitude transitional zone of Oromiya (Boran and Horro). Geographic coordinates and elevation information for the seven sampling populations were obtained from the original sample metadata and are summarized in Table 1. These elevation data were used to classify the populations according to predefined altitude thresholds. Based on sampling elevation, the seven Ethiopian indigenous cattle populations were classified into three elevation categories: low-altitude populations (<1000 m above sea level, masl), including Abigar (523 m), Barka, and Felata; mid-altitude populations (1000–3000 m masl), including Boran, Fogera, and Horro; and high-altitude populations (>3000 m masl), represented by Gojjam-Highland.

### 2.2. Variant Calling and Quality Assessment

Raw paired-end sequencing reads from 70 individuals were subjected to quality assessment and preprocessing. The initial sequencing quality was evaluated using FastQC v0.11.9, followed by adapter removal and quality trimming using Trimmomatic v0.39 [30]. The cleaned reads were aligned to the latest bovine reference genome ARS-UCD2.0 using BWA-MEM v0.7.17-r1188 [31]. The reference genome was downloaded from the NCBI Genome database (GCF_002263795.3_ARS-UCD2.0). All genomic analyses in this study were performed using the ARS-UCD2.0 reference assembly. Therefore, differences may exist between genome assembly versions used in this study and those reported in previous studies based on earlier assemblies (e.g., ARS-UCD1.2), including variations in assembly completeness, scaffold continuity, and genomic coordinate annotation. The adoption of ARS-UCD2.0 was intended to minimize mapping errors caused by limitations of previous assemblies and improve the accuracy of downstream variant detection. The resulting SAM files were converted into BAM format using SAMtools v1.19.2 [32] and sorted according to reference chromosome coordinates. PCR-duplicated reads were identified and marked using the MarkDuplicates function implemented in Picard v3.1.1. Indexed BAM files were subsequently generated using the samtools index command. Genomic variants were identified using GATK v4.5.0.0 [33]. The HaplotypeCaller module was applied to generate individual genomic variant information in GVCF format. To improve computational efficiency, variant calling was performed separately for each chromosome using the -L parameter. Individual GVCF files from all samples were merged by chromosome using CombineGVCFs, followed by joint genotyping using GenotypeGVCFs. Variant calls from all chromosomes were subsequently merged using MergeVcfs to generate the final multisample VCF dataset. Functional annotation of identified variants was performed using SnpEff v5.2a [34].

### 2.3. Population Genetic Analyses

Population genetic structure was investigated based on genome-wide SNP genotypes to characterize genetic differentiation among Ethiopian indigenous cattle populations distributed across different altitude categories. For population structure analyses, high-confidence biallelic autosomal SNPs were filtered using PLINK v1.9 [35] by removing variants with missing genotype rates > 5% and minor allele frequencies (MAF) < 5%. To minimize the potential influence of linkage disequilibrium (LD), the remaining SNPs were further pruned using a sliding window of 50 SNPs, a step size of 5 SNPs, and an r^2^ threshold of 0.2 (--indep-pairwise 50 5 0.2). After LD pruning, 1,435,022 approximately independent autosomal SNPs were retained for subsequent PCA and ADMIXTURE analyses. The VCF dataset was converted into PLINK-compatible formats using PLINK. Principal component analysis (PCA) was first performed on all 70 individuals using the LD-pruned SNP dataset, and eigenvectors of the first 10 principal components were calculated for each individual. PCA plots were generated using the ggplot2 v4.0.3 package in R v4.5.0. Population ancestry structure was further evaluated using ADMIXTURE v1.3.0 based on the same LD-pruned SNP dataset, with ancestral population numbers (K) ranging from 2 to 7. Model fit was evaluated using cross-validation error values, and ancestry proportions at higher K values were visualized for exploratory assessment of population ancestry patterns.

To evaluate whether cryptic relatedness influenced population structure inference, pairwise genomic relatedness was estimated using KING implemented in PLINK 2.0. Individuals involved in second-degree or closer relationships were filtered using a kinship coefficient cutoff of 0.0884. This procedure retained 57 relatively unrelated individuals. PCA and ADMIXTURE analyses were repeated using this relatedness-controlled subset as sensitivity analyses to evaluate whether the inferred population structure was robust to the potential influence of cryptic relatedness. Phylogenetic reconstruction was conducted separately using the original genome-wide SNP dataset. A total of 42,176,147 SNPs were used to calculate genetic distance matrices using VCF2Dis v1.51, and phylogenetic relationships were visualized using the ggtree v4.0.5 package in R.

Linkage disequilibrium (LD) analysis was subsequently performed to characterize the extent and decay patterns of non-random associations between SNP loci among different cattle populations. The VCF dataset was first filtered using BCFtools v1.19 by retaining only high-confidence variants with PASS status and biallelic SNPs. Population-specific VCF datasets were generated according to predefined population groups. Before LD estimation, additional quality control was conducted using VCFtools, and SNPs with minor allele frequencies (MAF) < 5% or missing genotype rates > 20% were removed. Only autosomal SNPs were retained for LD analysis to avoid potential bias caused by sex chromosomes. Pairwise LD was estimated independently for each population using PopLDdecay v3.45 based on the squared correlation coefficient (r^2^) between SNP pairs. SNP pairs within a maximum physical distance of 500 kb were considered for LD calculation. The average r^2^ values were calculated across different physical distance intervals, and LD decay curves were generated using the PopLDdecay plotting pipeline. Differences in LD decay patterns among populations were compared to infer variation in historical recombination patterns and demographic history.

### 2.4. Genome-Wide Scans for Selective Signatures

The original VCF dataset was filtered using GATK VariantFiltration with hard filtering criteria to remove low-quality variants. SNPs were marked as low quality if they met any of the following thresholds: read depth (DP) < 10, quality by depth (QD) < 2.0, Fisher strand bias (FS) > 60.0, strand odds ratio (SOR) > 4.0, mapping quality (MQ) < 40.0, mapping quality rank sum test (MQRankSum) < −12.5, or read position rank sum test (ReadPosRankSum) < −8.0. Variants passing all filtering criteria were retained for subsequent population genomic analyses. SNPs with missing genotypes > 20% were removed. Genome-wide nucleotide diversity (θπ) and fixation index (*F_ST_*) were calculated using VCFtools v0.1.15 [36]. Candidate genomic regions associated with altitude adaptation were identified by integrating genome-wide differentiation and nucleotide diversity divergence signals. Genome-wide selective sweep analyses were performed by comparing low-altitude populations with combined mid-to-high-altitude populations. The mid-to-high-altitude group included Boran, Fogera, Horro, and Gojjam-Highland populations. Genome-wide analyses were performed using a sliding-window approach with a window size of 500 kb and a step size of 50 kb. These parameters were chosen to balance statistical stability and genomic resolution: the relatively broad window reduces stochastic fluctuations in window-based *F_ST_* and nucleotide diversity estimates, whereas the 50 kb step provides densely overlapping windows for localizing genomic regions showing consistent selection signals. To quantify relative differences in nucleotide diversity between the two ecological groups, the nucleotide diversity ratio was calculated as π_MH_/π_L_, where π_MH_ and π_L_ represent nucleotide diversity in the mid-to-high-altitude and low-altitude populations, respectively. The ratio was subsequently log2-transformed as log_2_(π_MH_/π_L_) [37]. Genome-wide average nucleotide diversity was broadly comparable between the two groups, with the mid-to-high-altitude populations showing only slightly higher mean nucleotide diversity than the low-altitude populations (0.004251612 versus 0.004234635, respectively). Therefore, the log2-transformed nucleotide diversity ratio was used to characterize local relative differences in nucleotide diversity between the two ecological groups, rather than to infer a genome-wide reduction in nucleotide diversity in either group. Positive values of log_2_(π_MH_/π_L_) indicate relatively higher nucleotide diversity in the mid-to-high-altitude populations, whereas negative values indicate relatively lower nucleotide diversity.

Genomic windows within the top 5% of the genome-wide *F_ST_* distribution were considered regions showing pronounced population differentiation. In parallel, windows within the top 5% of the log_2_(π_MH_/π_L_) distribution represented regions showing the strongest relative elevation of nucleotide diversity in the mid-to-high-altitude populations compared with the low-altitude populations. Windows simultaneously falling within the upper 5% tails of both distributions were retained as candidate altitude-associated genomic regions for subsequent functional annotation and enrichment analyses. This empirical outlier-based approach was used to prioritize genomic regions displaying concordant patterns of pronounced population differentiation and local nucleotide-diversity contrast between the two altitude groups. Visualization was performed using the ggplot2 package in R.

### 2.5. Functional Annotation and Enrichment Analysis of Candidate Altitude-Associated Genomic Regions

To identify genomic regions potentially associated with high-altitude adaptation, overlapping regions among the top 5% *F_ST_* values and the top 5% log_2_(π_MH_/π_L_) values were selected as candidate selective sweep regions. Genomic windows falling within the top 5% of both the FST and θπ ratio distributions were intersected, and the overlapping genomic intervals were defined as candidate selective regions supported by both selection-scan metrics. Here, overlap refers to concordance between the FST and θπ analyses and does not imply that these regions were independently identified in every cattle population. Candidate regions were annotated using SnpEff v5.2a based on the bovine reference genome ARS-UCD2.0 obtained from the Ensembl database. All SNPs and genes located within candidate regions were extracted for downstream functional analyses. Functional enrichment analysis of candidate genes was performed using the KEGG pathway analysis implemented in R. KEGG pathway enrichment analysis was performed using the clusterProfiler package. Pathways with nominal *p* values < 0.05 were considered as candidate enriched pathways for subsequent biological interpretation. Because this analysis was used for exploratory prioritization of candidate genes derived from selective sweep regions, pathways were further evaluated based on their biological relevance to high-altitude adaptation and pathway connectivity [38].

### 2.6. In Silico Virtual Knockout Analysis of Candidate Genes

Single-cell RNA sequencing data used in this study were obtained from the Gene Expression Omnibus (GEO) database (GSE266061), including lung transcriptomic profiles from yak, cattle–yak hybrids, and cattle. Because Ethiopian indigenous cattle are classified within Bos taurus and breed-specific lung single-cell transcriptomic resources are currently unavailable, publicly available Bos taurus lung single-cell RNA-seq datasets were used as a reference framework for regulatory network perturbation analysis. Three biological replicates of cattle lung samples (GSM8240037–GSM8240039) were selected for analysis. Single-cell RNA-seq data processing was performed using R v4.5.0 and Seurat v5.0 [39]. RNA count layers from the three cattle lung samples were combined, yielding an expression matrix containing 35,676 genes and 36,547 cells, including 10,521, 15,349, and 10,677 cells from the three samples, respectively. Prior to virtual knockout analysis, genes detected in fewer than 5% of cells were removed, leaving 7272 genes. Genes with zero variance were subsequently excluded, although this step did not further reduce the number of retained genes. Because these filtering steps were performed at the gene level, all 36,547 cells were retained as the input cell pool for downstream analysis. Among the 1816 genes located within the candidate selective regions, candidate genes were further prioritized based on their recurrence across significantly enriched pathways relevant to high-altitude adaptation. In particular, we focused on five signaling pathways associated with hypoxia response, vascular regulation, and cellular stress adaptation, including the phospholipase D, Ras, ErbB, Wnt, and Hedgehog signaling pathways. *PIK3CA* and *RHOA* were each involved in multiple of these pathways and were therefore selected as representative high-priority candidate genes for subsequent virtual knockout analysis. Virtual knockout analyses of *PIK3CA* and *RHOA* were performed using the scTenifoldKnk package v1.0 [40]. Internal quality control was disabled (qc = FALSE) because the expression matrix had already been preprocessed. For each target gene, five gene regulatory networks were generated, with 500 cells subsampled from the 36,547-cell pool for each network (nc_nNet = 5, nc_nCells = 500). Network construction was performed using principal component regression with three principal components (nc_nComp = 3).

The regulatory connections associated with the target gene were removed by setting the corresponding adjacency matrix elements to zero, thereby simulating complete loss of gene regulatory activity and generating a pseudo-knockout network. Differences between wild-type and pseudo-knockout networks were quantified using quasi-manifold alignment. Genes exhibiting larger projection distances were considered more strongly affected by target gene perturbation and were defined as differentially regulated genes (DEGs). Statistical significance was determined using the criteria of adjusted *p* value < 0.05 and |log_2_FC| > 0.5. Functional enrichment analysis of DEGs was performed using clusterProfiler v4.10 [41,42], with all detected genes used as the background set. GO and KEGG enrichment analyses were performed using bovine annotation databases, with Benjamini–Hochberg correction applied and adjusted *p* < 0.05 considered statistically significant. Protein–protein interaction (PPI) information for upregulated hub genes was obtained from the STRING database v11.5 (combined score ≥ 400) [43]. Networks were constructed using the igraph v2.2.2, and hub nodes were identified based on node degree values exceeding the mean plus one standard deviation or ranking among the top 5% of nodes [44]. All analyses were conducted in the R statistical environment.

## 3. Results

### 3.1. SNP Identification and Functional Annotation

We performed whole-genome resequencing of 70 Ethiopian indigenous cattle individuals representing seven breeds distributed across three major agro-ecological zones of Ethiopia. The sequencing data exhibited high quality, with an average sequencing depth of 13.09× per individual. More than 90% of individuals achieved sequencing depths exceeding 11×, indicating sufficient genome-wide coverage and sequencing reliability (Supplementary Table S2). Following stringent variant filtering criteria, we identified a total of 49,196,624 high-confidence single-nucleotide polymorphisms (SNPs) across the 70 cattle genomes (Supplementary Table S3). Functional annotation using SnpEff revealed that the majority of identified variants were located in intergenic regions (48.11%) and intronic regions (37.13%) (Supplementary Table S4).

**Figure 1:**
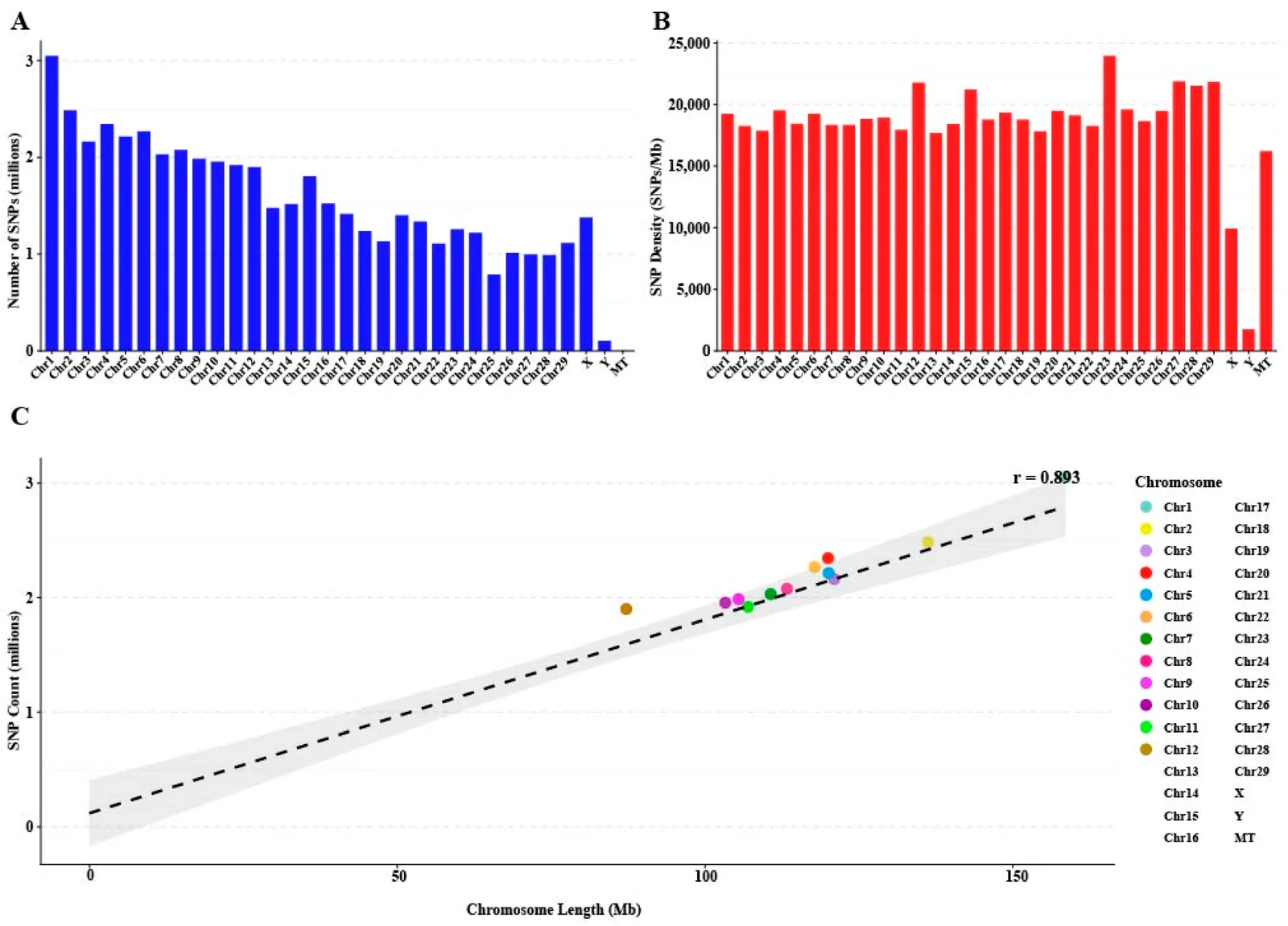
Genome-wide chromosomal distribution of SNPs. (**A**) Number of SNPs (millions) on each chromosome; (**B**) SNP density (SNPs/Mb) on each chromosome; (**C**) relationship between chromosome length and SNP count. Colored points represent individual chromosomes, the black dashed line represents the fitted linear regression, and the gray shaded band indicates the 95% confidence interval.

To further characterize the genomic distribution and potential functional effects of these genomic variants, all identified annotated variants were classified according to their genomic annotations. Variants located in upstream and downstream gene regions accounted for 8.30% and 3.62% of all SNPs, respectively, whereas coding-region variants, including synonymous and missense mutations, represented 1.11% of the total variants (Supplementary Table S5). Notably, functional annotation identified 20,711 frameshift variants (0.04%) and 2925 stop-gained variants among all annotated genomic variants, representing high-impact mutations that may contribute to breed-specific phenotypic variation. (Supplementary Table S5). Chromosome-level analysis revealed substantial variation in SNP density among chromosomes (Supplementary Table S6). The average SNP density across autosomes was 18.31 SNPs/kb, with chromosome NC_037350.1 exhibiting the highest SNP density (23.96 SNPs kb^−1^) and chromosome NC_082638.1 showing the lowest density (1.74 SNPs kb^−1^) (Supplementary Table S6). This chromosome-specific heterogeneity may reflect differences in recombination landscapes, selective pressures, and underlying genomic architectures. The X chromosome (NC_037357.1) exhibited a SNP density of 9.91 SNPs kb^−1^, markedly lower than the autosomal average, consistent with the reduced effective population size, hemizygous inheritance pattern, and distinct evolutionary dynamics of the X chromosome (Figure 1).

### 3.2. Population Structure, Genetic Relationships, and LD Decay 

**Figure 2:**
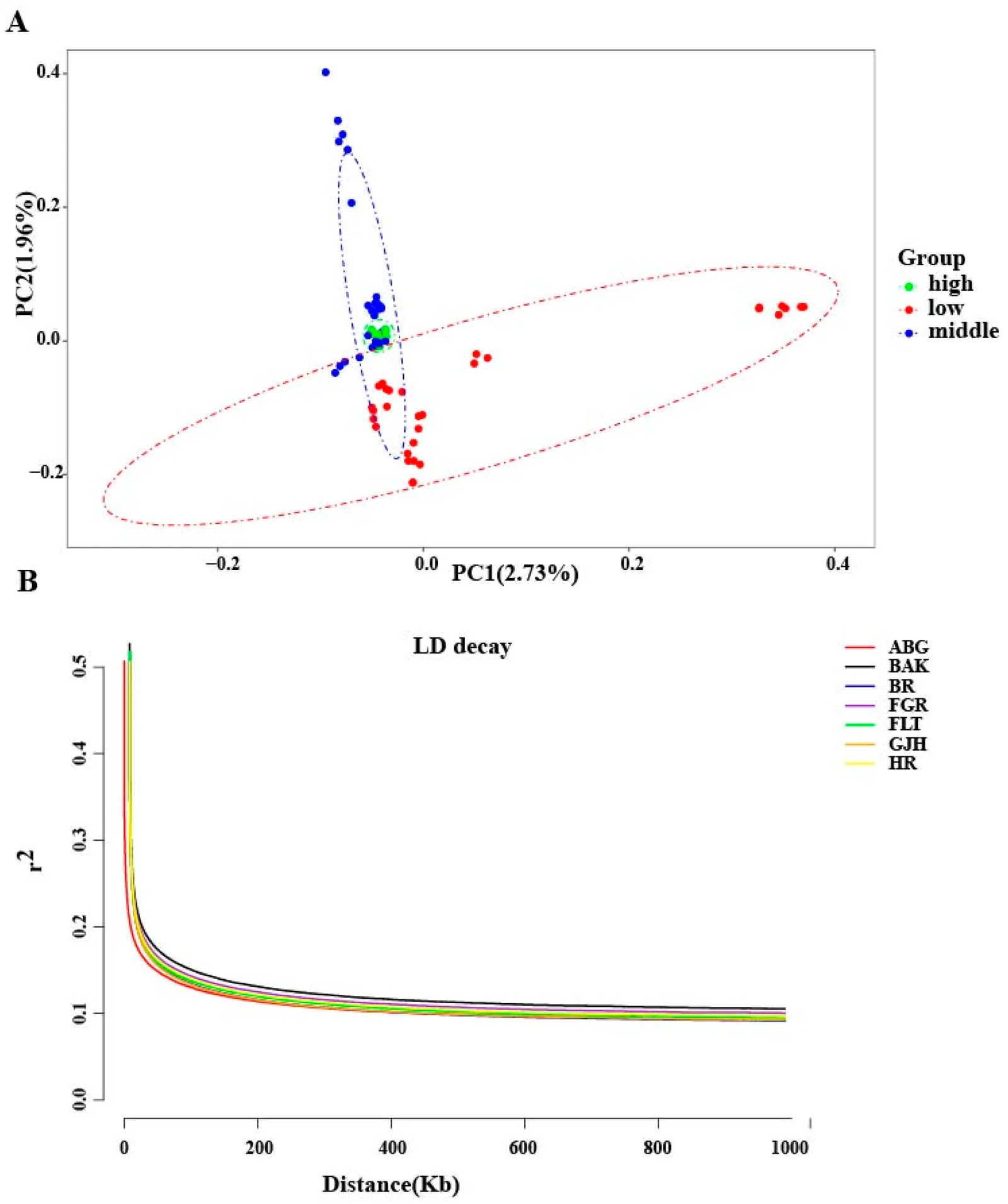
(**A**) Principal component analysis (PCA) of 70 Ethiopian indigenous cattle individuals based on genome-wide SNPs; (**B**) linkage disequilibrium (LD) decay patterns.

After LD pruning, 1,435,022 approximately independent autosomal SNPs were retained for population structure analyses. PCA based on all 70 individuals revealed pronounced genetic structure among the Ethiopian indigenous cattle populations (Figure 2A). Low-altitude populations were generally differentiated from many mid-to-high-altitude populations, although partial overlap among altitude categories remained. In particular, the high-altitude Gojjam-Highland population showed genetic proximity to several mid-altitude individuals, indicating that the observed clustering pattern could not be attributed to altitude alone. To assess the potential effect of cryptic relatedness, pairwise genomic relatedness was evaluated using KING. After excluding individuals involved in second-degree or closer relationships (kinship coefficient ≥ 0.0884), 57 relatively unrelated individuals were retained. PCA based on this relatedness-controlled subset showed an overall population structure broadly consistent with that obtained from the full LD-pruned dataset (Supplementary Figure S1), indicating that the major clustering pattern was not solely driven by close familial relationships. Nevertheless, breed-specific clustering and partial overlap among altitude categories remained evident, suggesting that the observed genomic structure likely reflects the combined effects of breed background, geographic history, and environmental differentiation. Therefore, subsequent selective sweep analyses were interpreted as identifying candidate altitude-associated regions rather than proving altitude-specific selective events.

Linkage disequilibrium (LD) decay patterns were assessed across seven Ethiopian indigenous cattle populations based on the squared correlation coefficient (r^2^) between SNP pairs. All populations exhibited a typical LD decay pattern, characterized by a rapid decrease in r^2^ with increasing physical distance between markers, followed by gradual stabilization at longer distances (Figure 2B), indicating the progressive erosion of linkage associations due to historical recombination events. Among the studied populations, BAK showed consistently higher LD levels and slower LD decay compared with other populations, indicating a relatively stronger persistence of linkage associations. FGR also exhibited relatively elevated LD levels, whereas ABG, BR, FLT, GJH, and HR showed similar LD decay patterns with lower r^2^ values at longer genomic distances. The differences in LD decay patterns among populations may reflect variation in demographic history and historical recombination processes among Ethiopian indigenous cattle populations.

**Figure 3:**
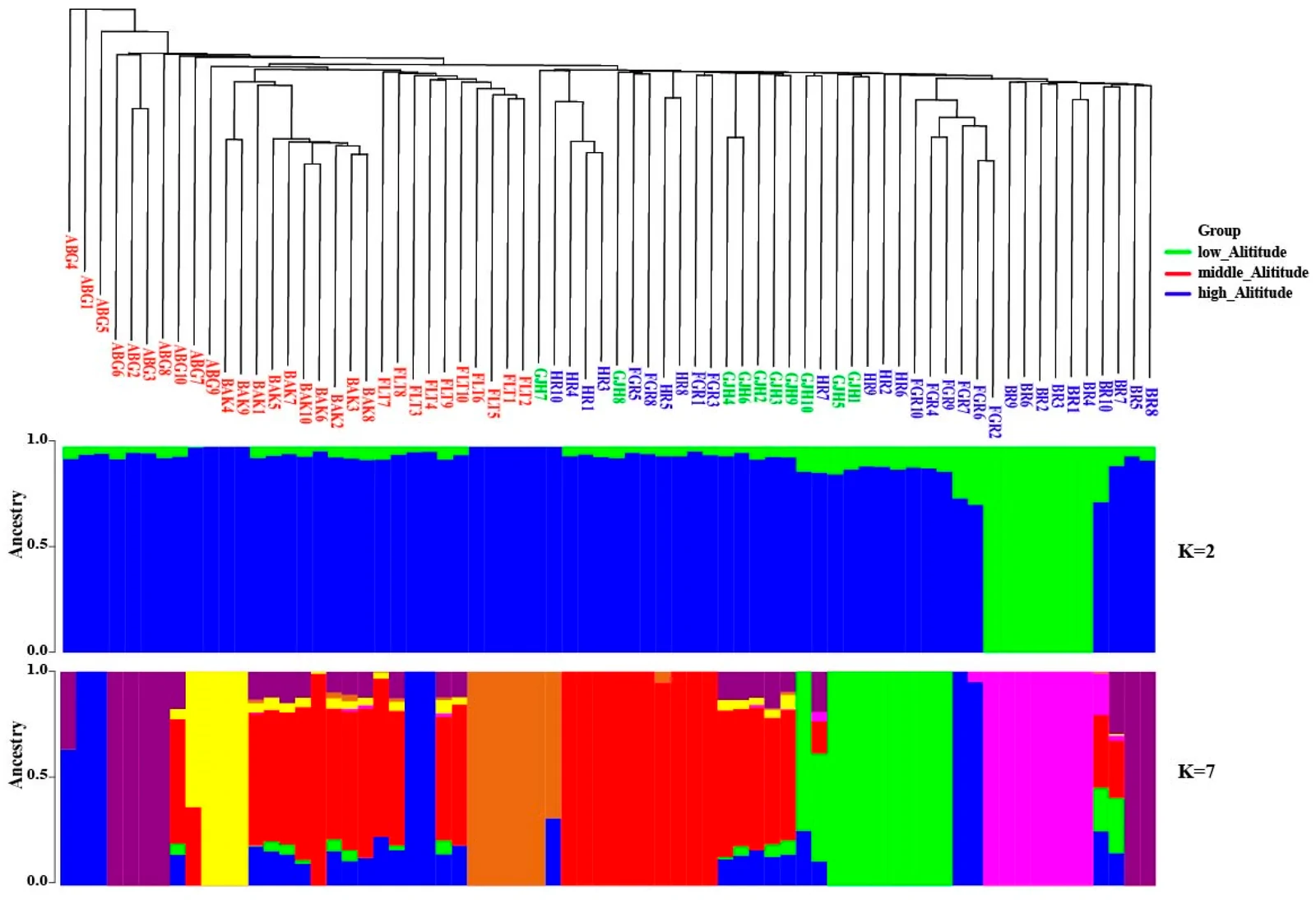
Phylogenetic relationships and population ancestry structure of Ethiopian indigenous cattle. In the phylogenetic tree, tip-label colors indicate altitude groups (low, middle, and high). In the ADMIXTURE plots, each vertical bar represents an individual, and different colors represent inferred ancestral components at K = 2 and K = 7.

Phylogenetic reconstruction broadly supported the presence of population-level genetic differentiation observed in the PCA. Low-altitude cattle formed a distinct major lineage, whereas several mid-to-high-altitude populations, including BR, FGR, GJH, and HR, showed closer phylogenetic relationships. However, individuals within the mid-to-high-altitude populations were partially intermingled, indicating that their genetic relationships were not strictly structured according to altitude category alone. ADMIXTURE analysis based on the LD-pruned autosomal SNP dataset further characterized ancestry variation among the seven cattle populations (Figure 3; Supplementary Figure S2). At K = 2, differentiation in ancestry composition was observed among the sampled populations, whereas increasing K values revealed progressively finer population- and breed-associated ancestry patterns. Considerable ancestry sharing was observed among several mid- and high-altitude populations, consistent with their relatively close genetic relationships indicated by PCA and phylogenetic analyses. Complete ADMIXTURE profiles from K = 2 to K = 7 are provided in Supplementary Figure S2. ADMIXTURE analysis was further repeated using the relatedness-controlled subset of 57 individuals. Population-associated ancestry patterns remained evident after the removal of closely related individuals, although the ancestry proportions of some populations changed following the reduction in sample size. Complete ADMIXTURE profiles for the 57-individual dataset from K = 2 to K = 7 are provided in Supplementary Figure S3. These results indicate that the major ancestry patterns observed in the full dataset were not solely attributable to close familial relationships.

### 3.3. Altitude-Associated Selective Signatures

**Figure 4:**
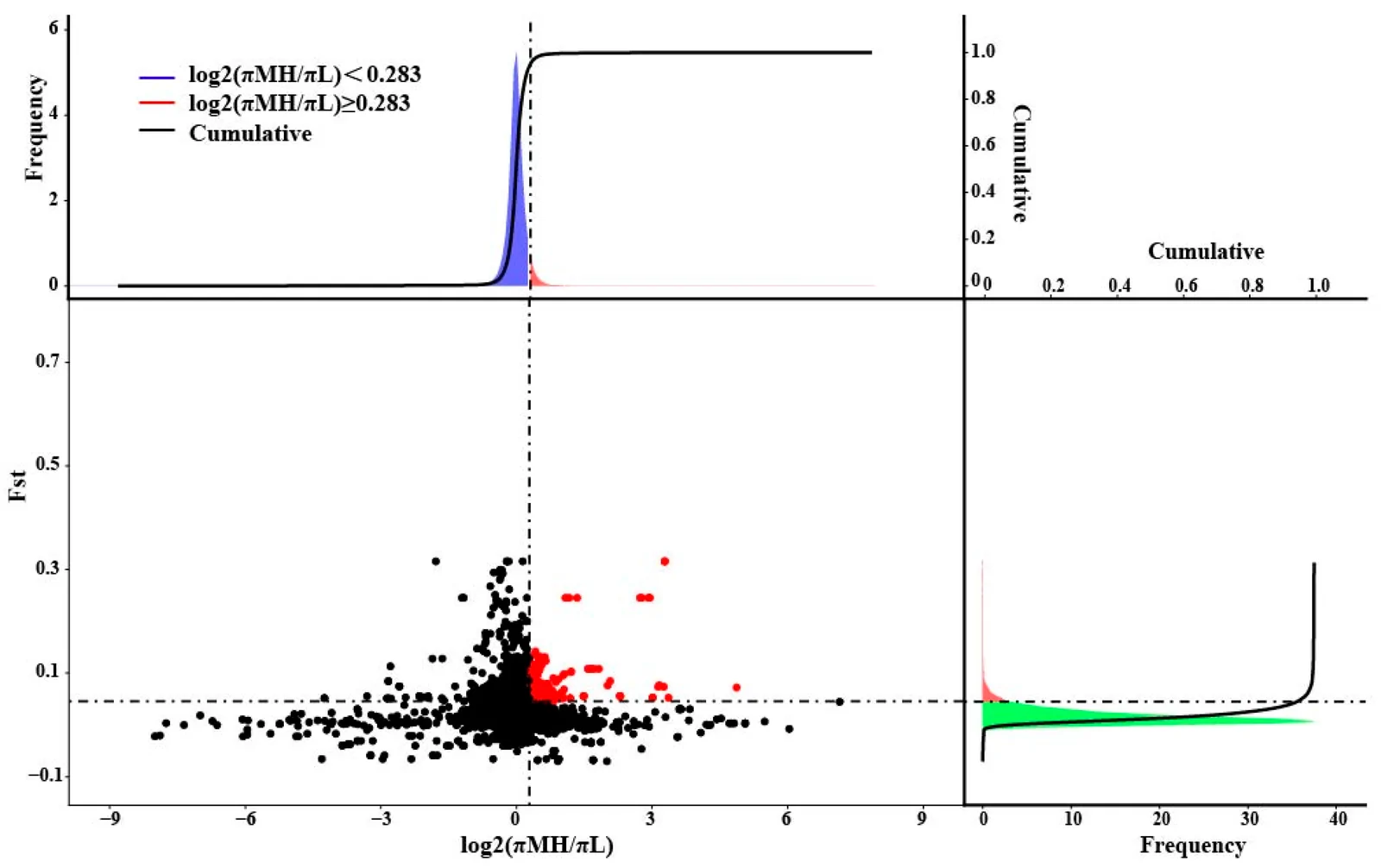
Genome-wide distribution of *F_ST_* and log_2_(π_MH_/π_L_) values. Red dots indicate genomic windows that simultaneously fall within the top 5% of both FST and θπ ratio distributions and were therefore considered candidate selective regions. Black dots represent genomic windows not meeting both selection thresholds, whereas red dots indicate windows simultaneously falling within the top 5% of both statistics. Blue/red shaded marginal distributions indicate windows below/above the log2(π_MH_/π_L_) cutoff, and green/red shaded distributions indicate windows below/above the FST cutoff. Black curves show cumulative distributions, and dashed lines indicate the empirical top-5% thresholds.

To identify genomic regions potentially associated with high-altitude adaptation, we calculated genome-wide fixation index (*F_ST_*) and nucleotide diversity ratios (θπ_MH_/θπ_L_) between mid-to-high-altitude and low-altitude populations using a sliding-window approach (500 kb windows with 50 kb steps; Supplementary Tables S7–S10). Genome-wide distributions of *F_ST_* and log_2_(π_MH_/π_L_) values revealed that most genomic windows exhibited relatively low differentiation, whereas a limited number of regions showed simultaneously elevated *F_ST_* and log_2_(π_MH_/π_L_) signals (highlighted as red dots; 398 in total, Supplementary Figure S4), indicating strong differentiation between high- and low-altitude populations and increased nucleotide diversity in high-altitude cattle populations. Candidate altitude-associated regions were identified by selecting genomic windows within the top 5% of both *F_ST_* and log_2_(π_MH_/π_L_) values. The use of overlapping signals from two independent statistics was intended to increase confidence in the identified regions by reducing potential false positives associated with individual selection metrics. The overlapping regions between these two independent approaches were subsequently used for downstream analyses (Figure 4; Supplementary Figure S4).

**Figure 5:**
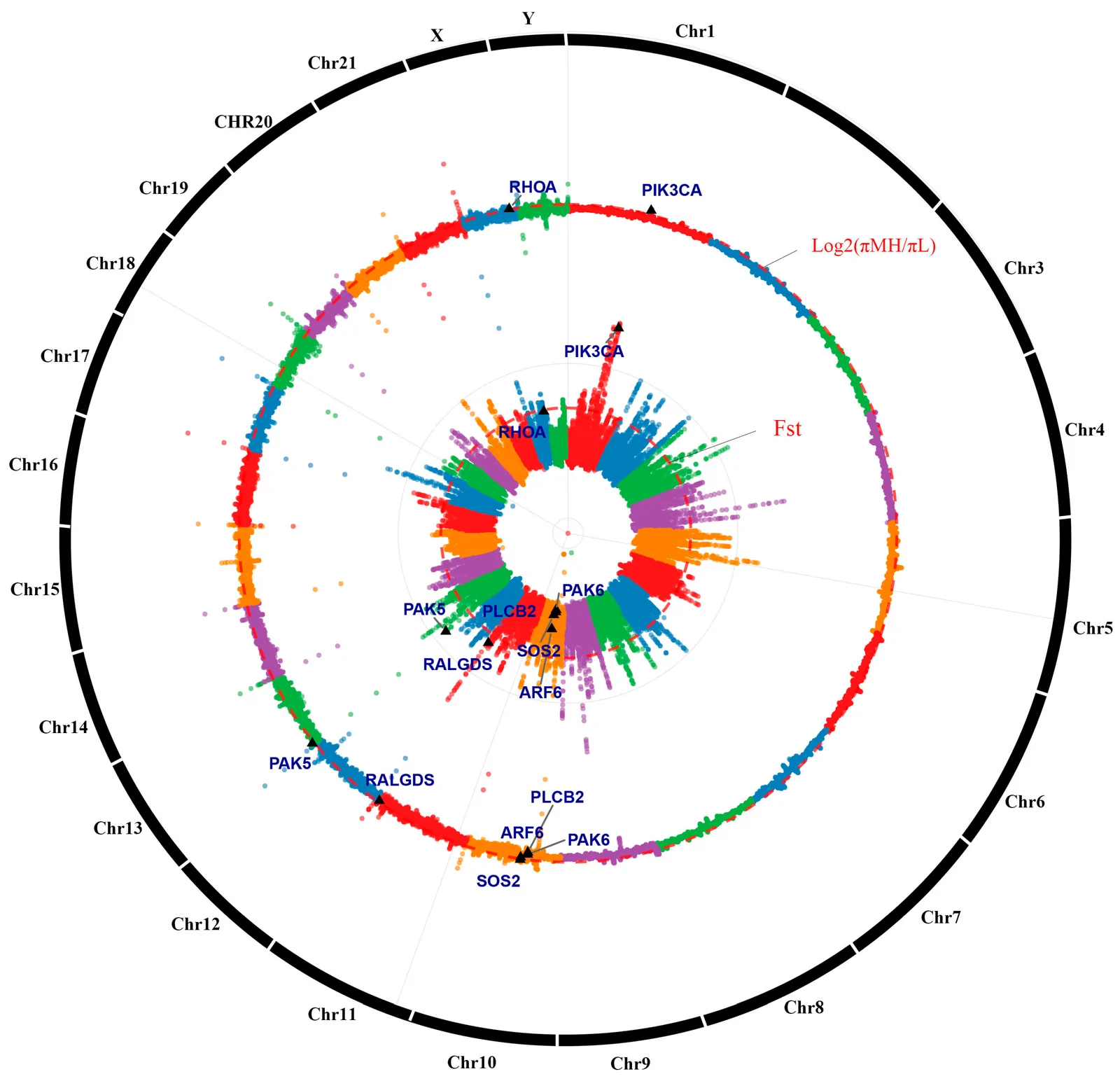
Identification of genomic regions associated with high-altitude adaptation in Ethiopian indigenous cattle based on the intersection of the top 5% *F_ST_* and log_2_(π_MH_/π_L_) signals. Representative candidate genes within the overlapping selective regions, including *PIK3CA*, *RHOA*, *PAK5*, *RALGDS*, *PLCB2*, *SOS2*, *ARF6*, and *PAK6* et al., are labeled in the figure. The complete list of candidate genes is provided in Supplementary Table S11. Different colors in the circular tracks distinguish chromosomes; the outer track represents log_2_(π_MH_/π_L_), the inner track represents FST, and the labeled markers indicate representative candidate genes.

Candidate windows displayed non-random distributions across chromosomes, with multiple chromosomal regions exhibiting strong enrichment of both *F_ST_* and log_2_(π_MH_/π_L_) signals. By intersecting the candidate regions identified by the two approaches, we obtained 398 shared candidate altitude-associated genomic regions. These regions contained genes potentially involved in adaptation to high-altitude environments, several key candidate genes were identified, including *PIK3CA*, *RHOA*, *PAK5*, *RALGDS*, *PLCB2*, *SOS2*, *ARF6*, and *PAK6*, providing potential targets for understanding the genetic basis of high-altitude adaptation in Ethiopian indigenous cattle (Figure 5; Supplementary Table S11).

### 3.4. Functional Enrichment Analysis and Candidate Genes Associated with High-Altitude Adaptation

**Figure 6:**
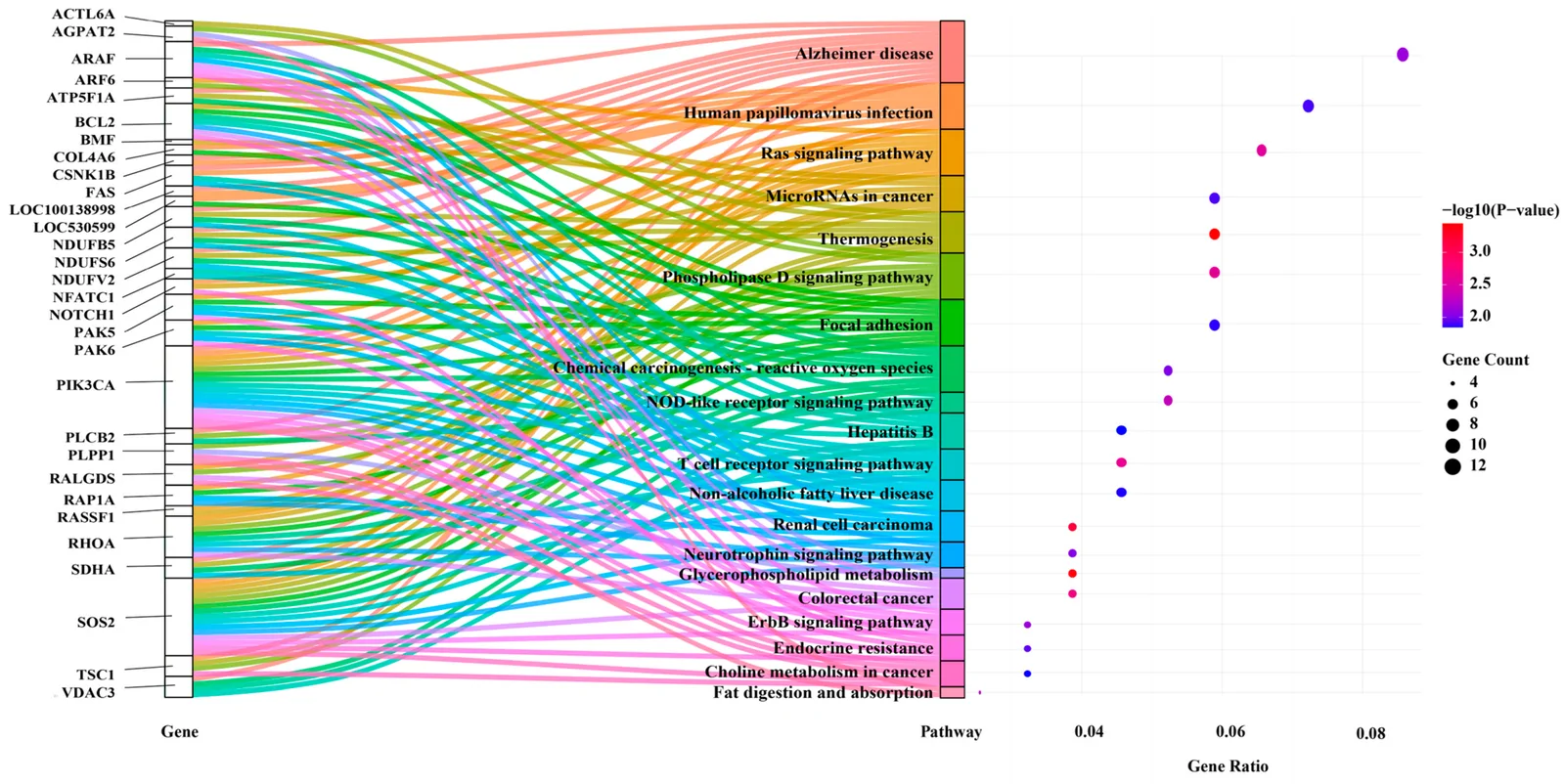
KEGG pathway enrichment analysis of candidate genes associated with high-altitude adaptation in Ethiopian indigenous cattle. The left panel shows gene–pathway relationships. In the right dot plot, dot size represents gene count, dot color represents −log10(*p* value), and the x-axis represents gene ratio.

The 398 candidate selective regions were annotated using SnpEff, resulting in the identification of 1816 genes located within these genomic intervals. KEGG pathway enrichment analysis of these candidate genes KEGG enrichment analysis identified 263 pathways showing nominal enrichment signals (*p* < 0.05) (Supplementary Table S11). Among these pathways, five pathways associated with hypoxia response, vascular regulation, and cellular stress adaptation were prioritized for further analysis., with the top 20 pathways shown in Figure 6. Among these enriched pathways, five major signaling pathways associated with hypoxia response, vascular regulation, and cellular stress adaptation were identified: Phospholipase D signaling pathway (*AGPAT2*, *ARF6*, *PIK3CA*, *PLCB2*, *PLPP1*, *RALGDS*, *RHOA*, *SOS2*, *TSC1*), Ras signaling pathway (*ARF6*, *PAK5*, *PAK6*, *PIK3CA*, *RALBP1*, *RALGDS*, *RAP1A*, *RASSF1*, *RHOA*, *SOS2*), ErbB signaling pathway (*ARAF*, *PAK5*, *PAK6*, *PIK3CA*, *SOS2*), Wnt signaling pathway (*CSNK1B*, *DKK4*, *LOC100138998*, *NFATC1*, *NKD2*, *PLCB2*, *RHOA*), Hedgehog signaling pathway (*BCL2*, *CSNK1B*, *LOC100138998*). To prioritize candidate genes for functional investigation, we further integrated the KEGG enrichment results with gene occurrence frequency within the selected candidate regions. Among all annotated genes located within the 398 candidate selective regions supported by both FST and θπ signals, we ranked genes according to their involvement in the five high-altitude-related signaling pathways (Phospholipase D, Ras, ErbB, Wnt, and Hedgehog pathways). Candidate pathways were prioritized based on their known biological relevance to high-altitude adaptation, particularly pathways involved in hypoxia response, vascular regulation, cellular stress response, and tissue remodeling. Genes repeatedly represented across these biologically relevant pathways were further prioritized according to pathway connectivity and occurrence frequency. Based on the convergence of selective sweep signals, biological relevance of enriched pathways, and pathway connectivity, *PIK3CA* and *RHOA* were selected as representative candidate hub genes for subsequent virtual knockout analyses because they participated in multiple enriched pathways and occupied central positions within the corresponding signaling networks.

### 3.5. In Silico Virtual Knockout Analysis Predicts Potential Regulatory Effects of PIK3CA and RHOA in High-Altitude Adaptation

**Figure 7:**
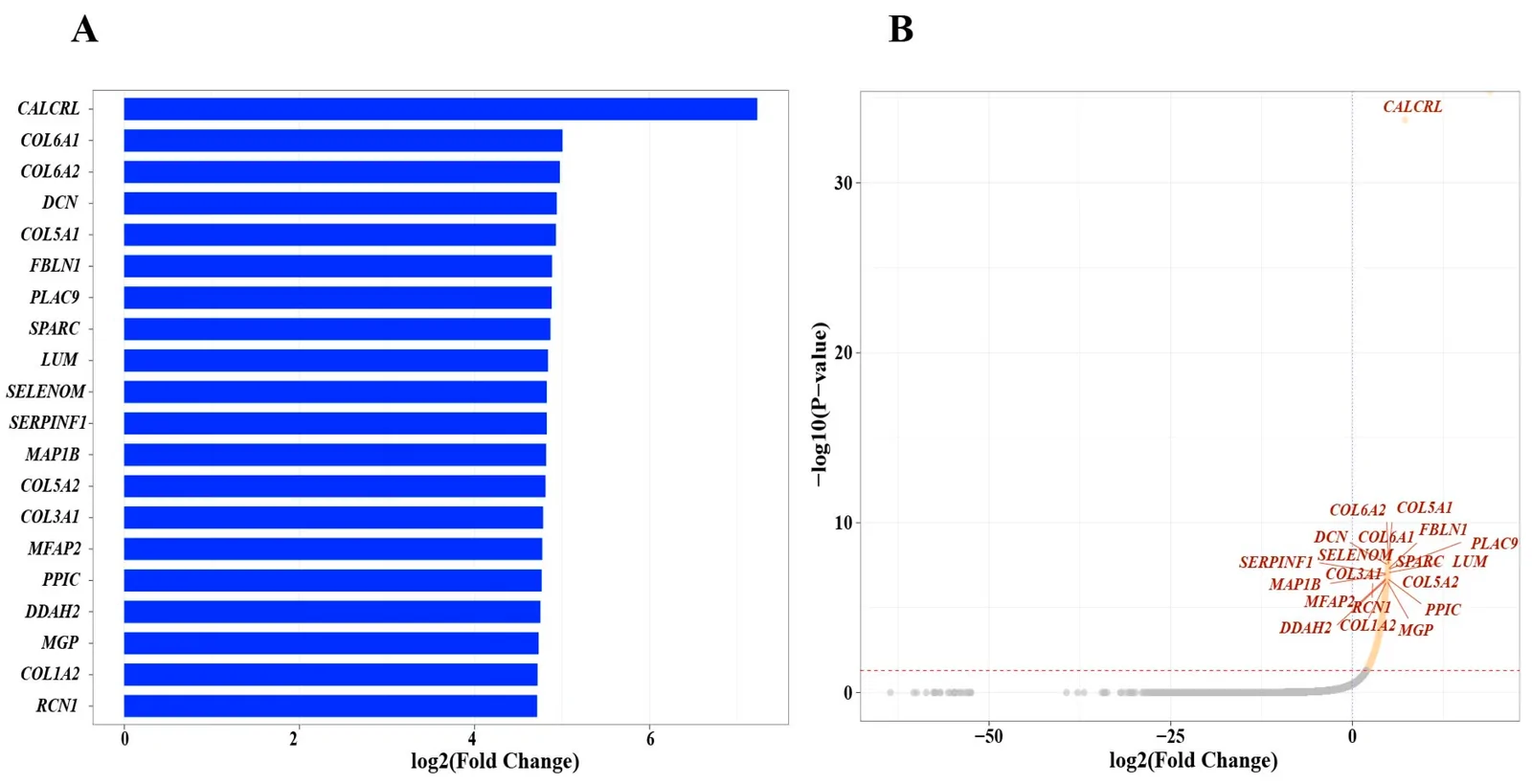
Differentially regulated genes following *PIK3CA* virtual knockout. (**A**) Top 20 genes predicted to increase following perturbation genes; (**B**) volcano plot of differentially regulated genes. In panel B, gray dots indicate non-significant genes, whereas orange dots indicate significant differentially regulated genes meeting adjusted *p* < 0.05 and log_2_(Fold Change).

In silico perturbation analysis of *PIK3CA* revealed a predominantly positive predicted transcriptional response following simulated gene disruption. Ranking the top 20 differentially regulated genes according to predicted log_2_ fold change (log_2_FC), *CALCRL* exhibited the strongest predicted increase (log_2_FC > 7), followed by *COL6A1*, *COL6A2*, *DCN*, *COL5A1*, *FBLN1*, *PLAC9*, *SPARC*, *LUM*, and *SERPINF1* (Supplementary Table S12). Volcano plot analysis further showed that most predicted differentially regulated genes were distributed in the positive log_2_FC region, indicating that these genes were predicted to increase following computational perturbation of *PIK3CA* (Figure 7B). Using the criteria of adjusted *p* < 0.05 and |log_2_FC| > 0.5, a set of significantly altered genes was identified, among which *CALCRL* showed the strongest predicted transcriptional response.

**Figure 8:**
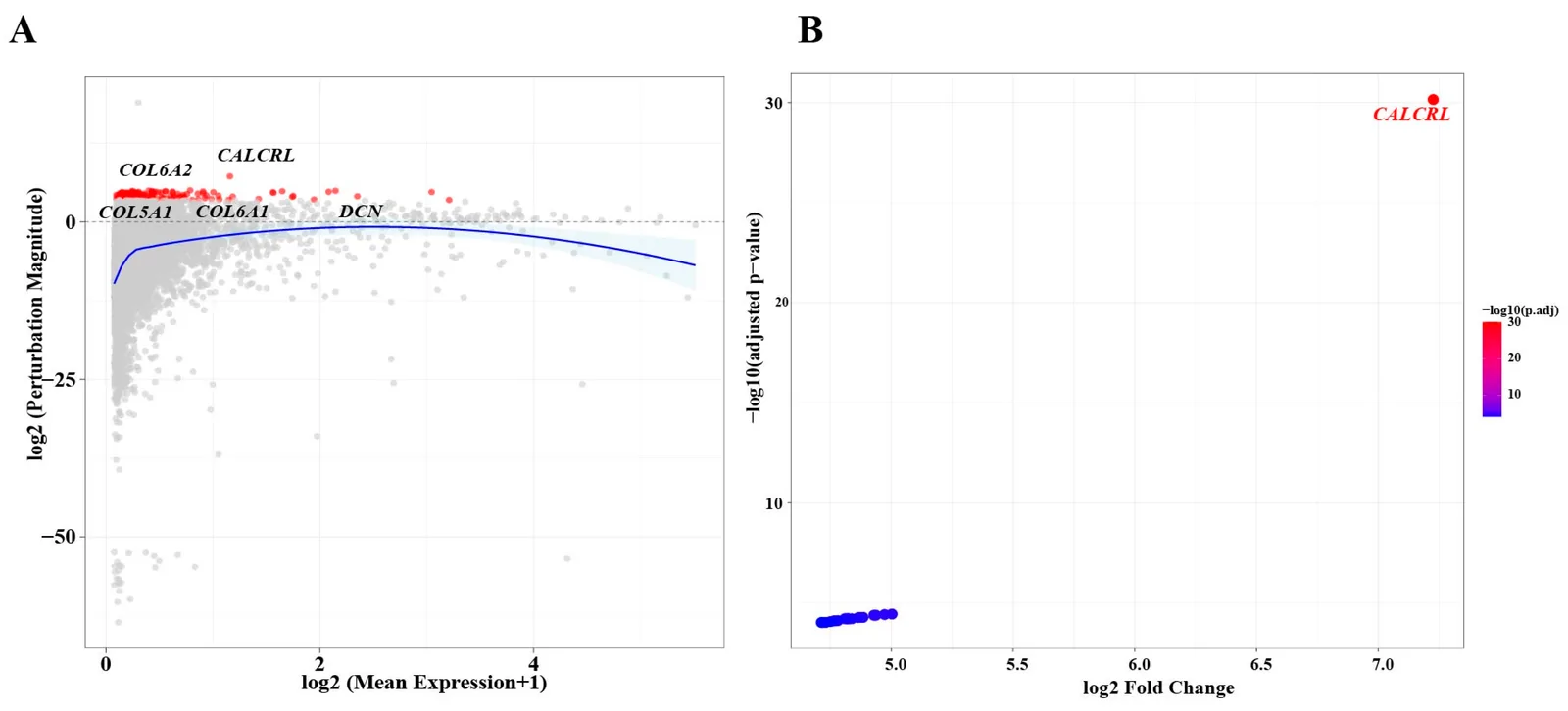
Perturbation–expression association analysis following in silico *PIK3CA* perturbation. (**A**) Scatter plot showing the relationship between predicted perturbation magnitude and average gene expression level. Red dots indicate predicted differentially regulated genes, whereas gray dots indicate non-significant genes. (**B**) Volcano plot showing predicted transcriptional changes following in silico *PIK3CA* perturbation, with predicted log_2_ fold change (log_2_FC) plotted against −log10(adjusted *p* value). The dashed line indicates zero perturbation magnitude; the blue fitted curve represents the overall trend and the shaded band represents the confidence interval.

The perturbation–expression association analysis following in silico *PIK3CA* perturbation showed that predicted differentially regulated genes (red dots) generally exhibited positive perturbation magnitudes (log_2_ Perturbation Magnitude > 0), whereas non-significant genes displayed broader distributions across expression levels (Figure 8A). Several genes, including *CALCRL*, *COL6A1*, *COL6A2*, and *DCN*, showed relatively high average expression levels and strong predicted perturbation responses, indicating their prominent responses within the computational perturbation analysis. Volcano plot analysis further demonstrated that these genes exhibited large positive predicted log_2_FC values and high statistical significance, with *CALCRL* representing one of the genes showing the strongest predicted transcriptional responses (Figure 8B).

**Figure 9:**
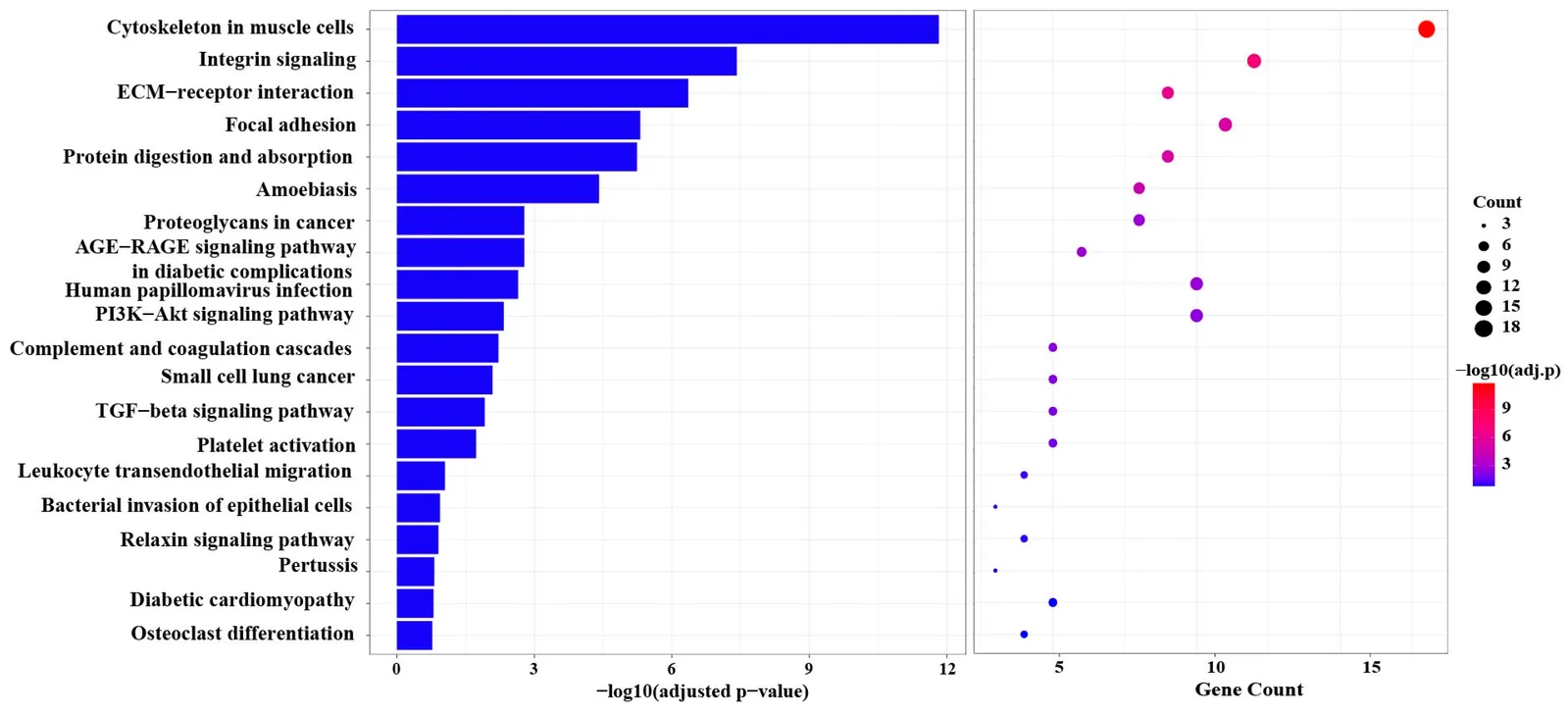
KEGG pathway enrichment analysis of genes affected by *PIK3CA* virtual knockout.

KEGG enrichment analysis of genes affected by *PIK3CA* perturbation revealed significant enrichment of pathways associated with cellular architecture, extracellular matrix regulation, and signal transduction (Figure 9; Supplementary Table S13). The major enriched pathways included Cytoskeleton in muscle cells, Integrin signaling pathway, ECM–receptor interaction, Focal adhesion, Protein digestion and absorption, Proteoglycans in cancer, PI3K–Akt signaling pathway, TGF-beta signaling pathway, Platelet activation, and Leukocyte transendothelial migration.

**Figure 10:**
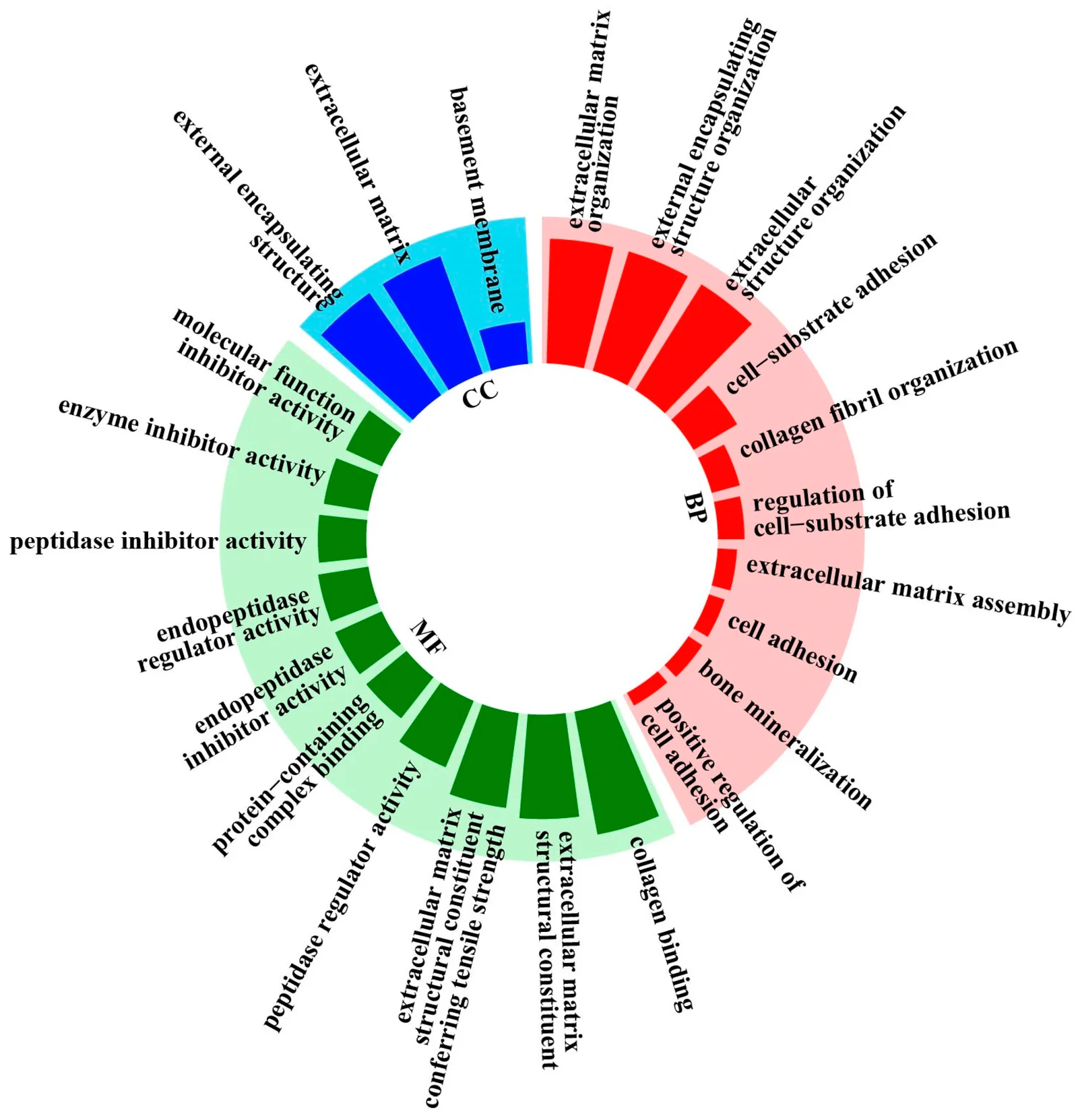
GO enrichment analysis of genes affected by *PIK3CA* virtual knockout. Red, blue, and green sectors represent biological process (BP), cellular component (CC), and molecular function (MF) categories, respectively.

GO enrichment analysis further demonstrated that *PIK3CA* perturbation was associated with transcriptional changes enriched in extracellular matrix organization and cell adhesion-related pathways within the integrated bovine lung transcriptomic network (Figure 10; Supplementary Tables S14–S16). At the biological process (BP) level, affected genes were significantly enriched in extracellular matrix organization, cell adhesion, collagen fibril organization, regulation of cell-substrate adhesion, and bone mineralization. Cellular component (CC) analysis showed enrichment in extracellular matrix, basement membrane, and external encapsulating structure, whereas molecular function (MF) categories were mainly associated with collagen binding, protein-containing complex binding, and peptidase regulator activity.

**Figure 11:**
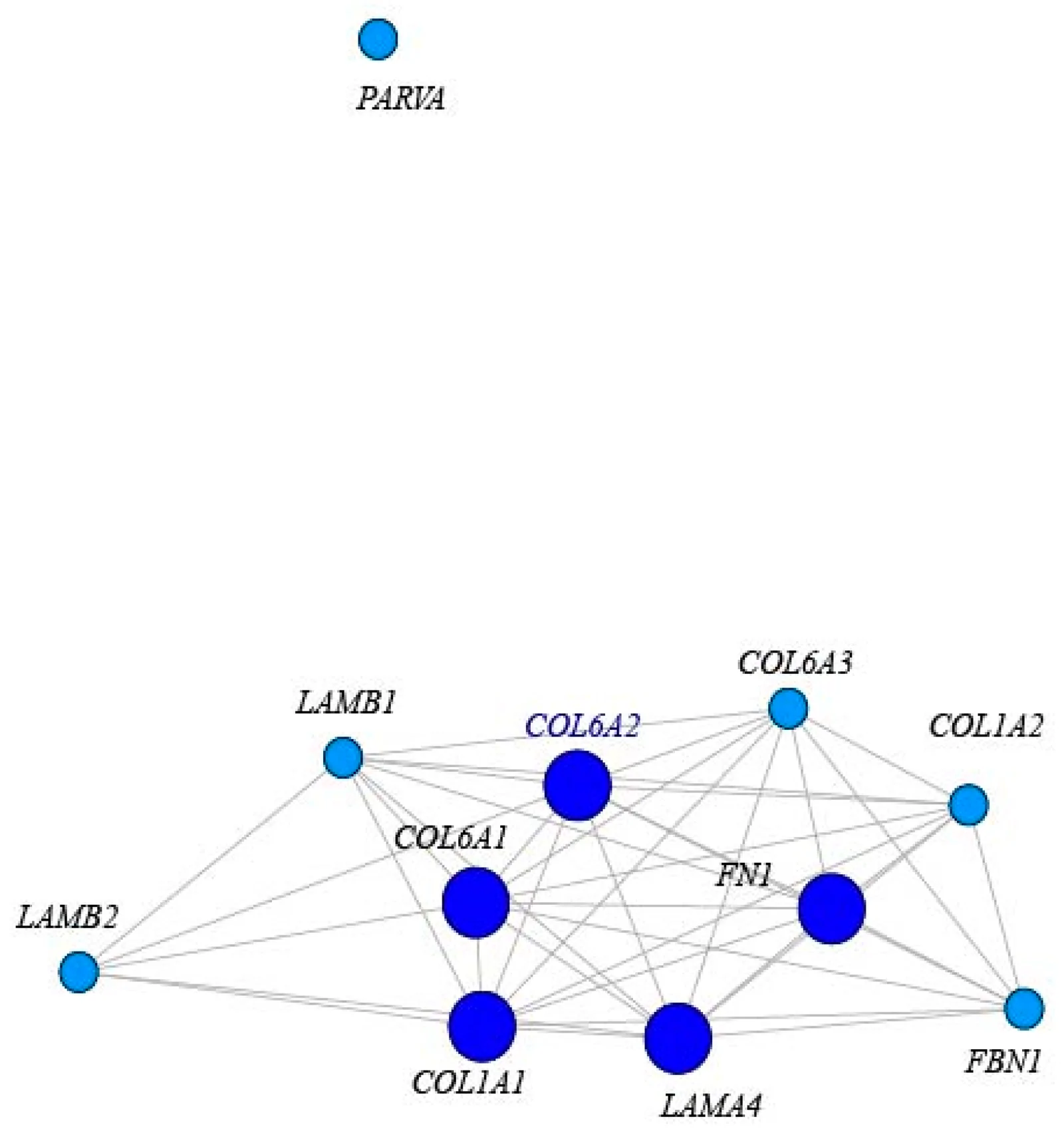
Protein–protein interaction network of genes affected by *PIK3CA* virtual knockout. Node size and color intensity represent node degree, with larger and darker nodes indicating higher connectivity; gray edges represent protein–protein interactions. Blue-highlighted gene labels indicate hub genes.

Protein–protein interaction (PPI) network analysis revealed a highly connected network consisting of 11 nodes and 40 edges (combined score ≥ 400) among *PIK3CA*-associated differentially regulated genes (Figure 11). Network topology analysis identified *COL1A1*, *COL1A2*, *COL6A1*, *COL6A2*, *LAMA4*, *FN1* and *FBN1* as central hub genes with high connectivity and network centrality, suggesting their potential roles in *PIK3CA*-associated extracellular matrix-related transcriptional changes.

**Figure 12:**
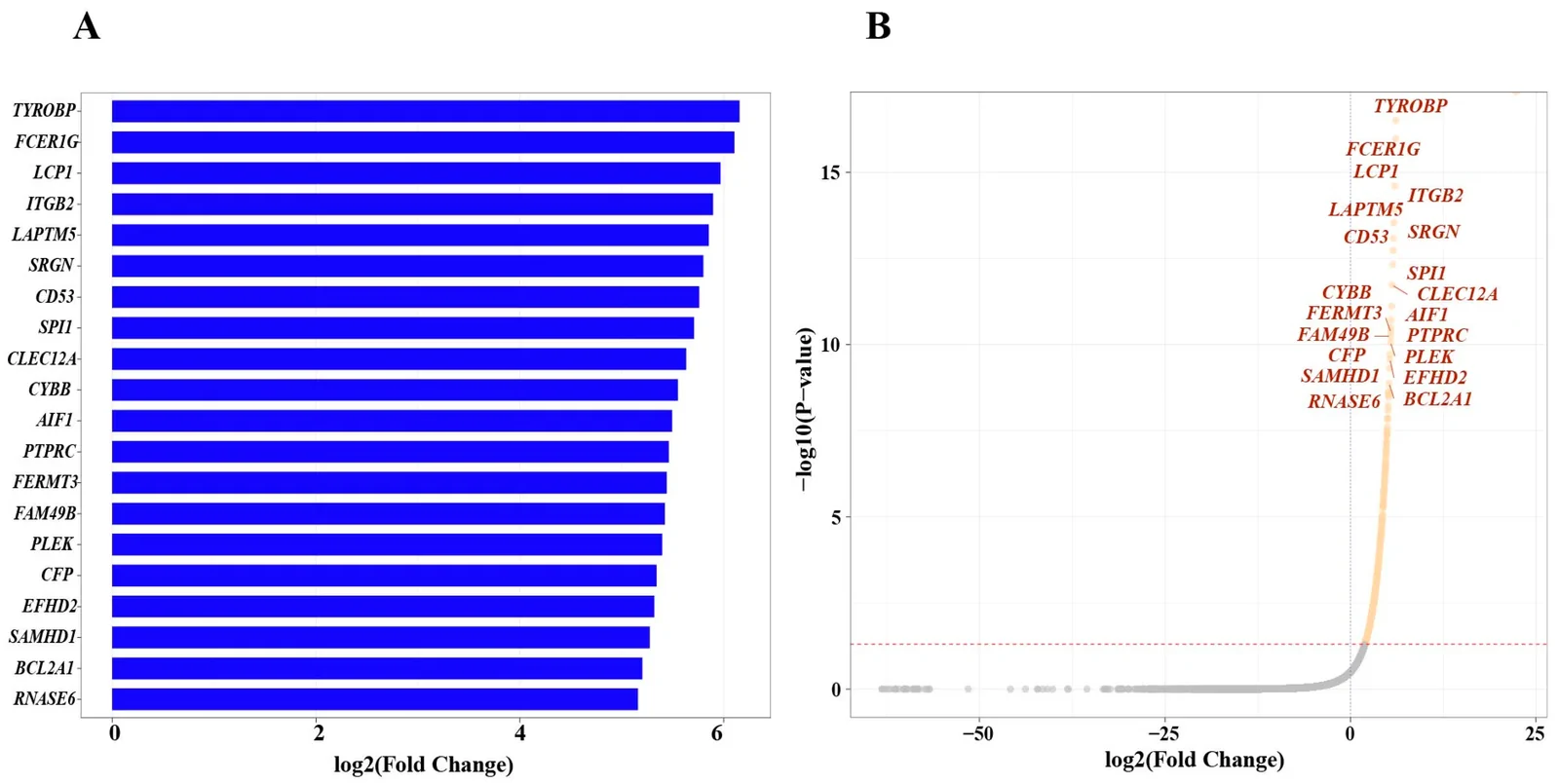
Differentially regulated genes following *RHOA* virtual knockout. (**A**) Top 20 genes predicted to increase following virtual perturbation; (**B**) volcano plot of differentially regulated genes. Gray dots indicate non-significant genes and orange dots indicate significant genes; the vertical dashed line indicates log_2_(Fold Change) = 0, and the horizontal dashed line indicates the statistical significance threshold.

In silico perturbation analysis of *RHOA* generated a distinct predicted transcriptional response characterized by enrichment of immune-associated gene programs. Among the top 20 differentially regulated genes, genes showing the strongest predicted increases included *TYROBP*, *FCER1G*, *LCP1*, *ITGB2*, *LAPTM5*, *SRGN*, *CD53*, *SPI1*, *CLEC12A*, *CYBB* and *AIF1*, which displayed strong enrichment of immune cell-associated signatures (Figure 12A; Supplementary Table S17). Volcano plot analysis demonstrated that *RHOA* perturbation predominantly resulted in genes with positive predicted log_2_FC values, suggesting that computational disruption of *RHOA* was associated with predicted changes in immune-related transcriptional programs (Figure 12B).

**Figure 13:**
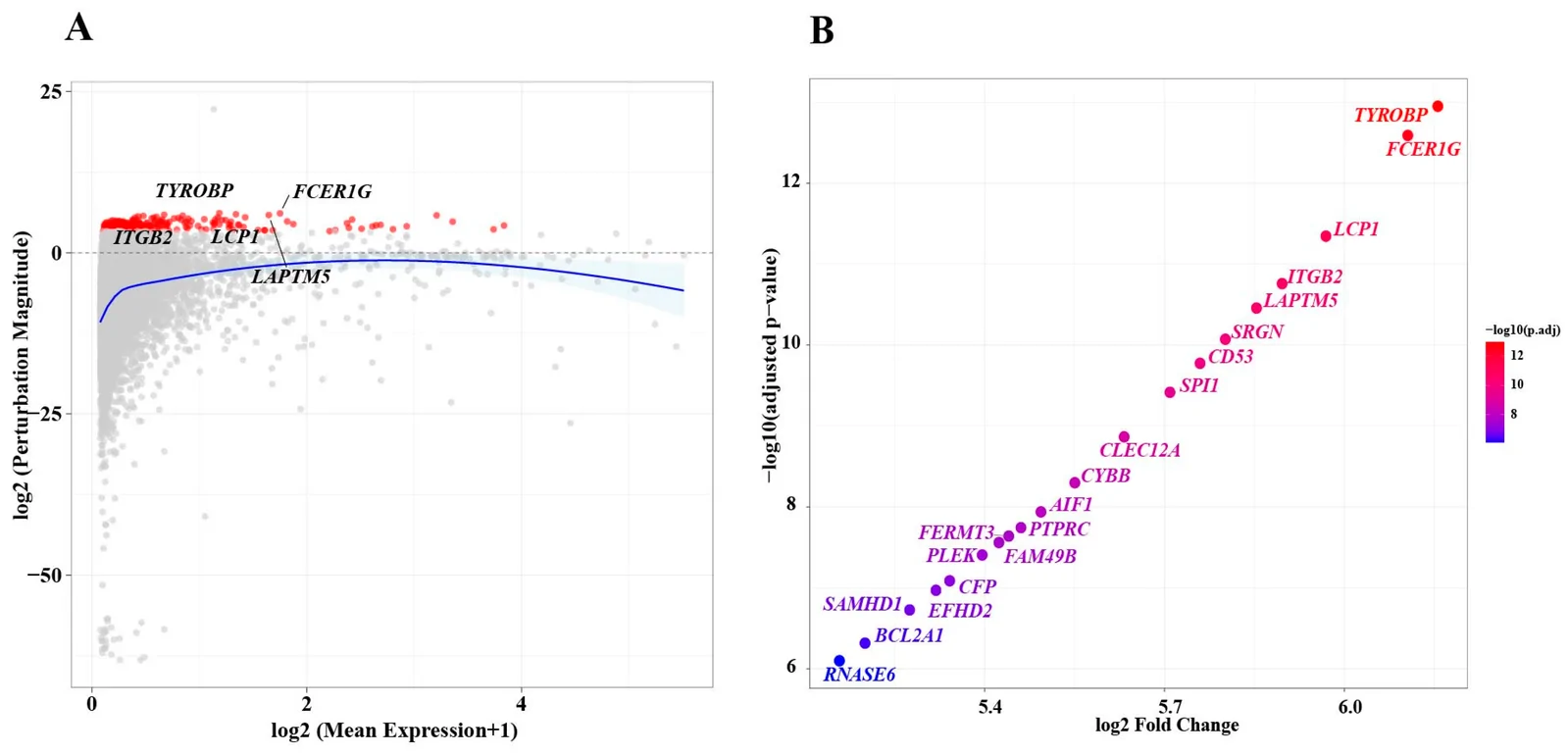
Perturbation–expression association analysis following in silico *RHOA* perturbation. (**A**) Scatter plot showing the relationship between predicted perturbation magnitude and average gene expression level. Red dots indicate predicted differentially regulated genes, whereas gray dots indicate non-significant genes. (**B**) Volcano plot showing predicted transcriptional changes following in silico *RHOA* perturbation, with predicted log_2_ fold change (log_2_FC) plotted against −log10(adjusted *p* value). The dashed line indicates zero perturbation magnitude; the blue fitted curve represents the overall trend and the shaded band represents the confidence interval.

Perturbation–expression association analysis following in silico *RHOA* perturbation showed that predicted differentially regulated genes exhibited predominantly positive perturbation magnitudes (Figure 13A). Genes including *TYROBP*, *FCER1G*, *LCP1* and *ITGB2* displayed relatively high average expression levels and strong predicted perturbation responses, indicating their prominent responses within the computational perturbation analysis. Volcano plot analysis demonstrated that these genes exhibited large positive predicted log_2_FC values and high statistical significance, with *TYROBP*, *FCER1G* and *LCP1* among the genes showing the strongest predicted transcriptional responses (Figure 13B).

**Figure 14:**
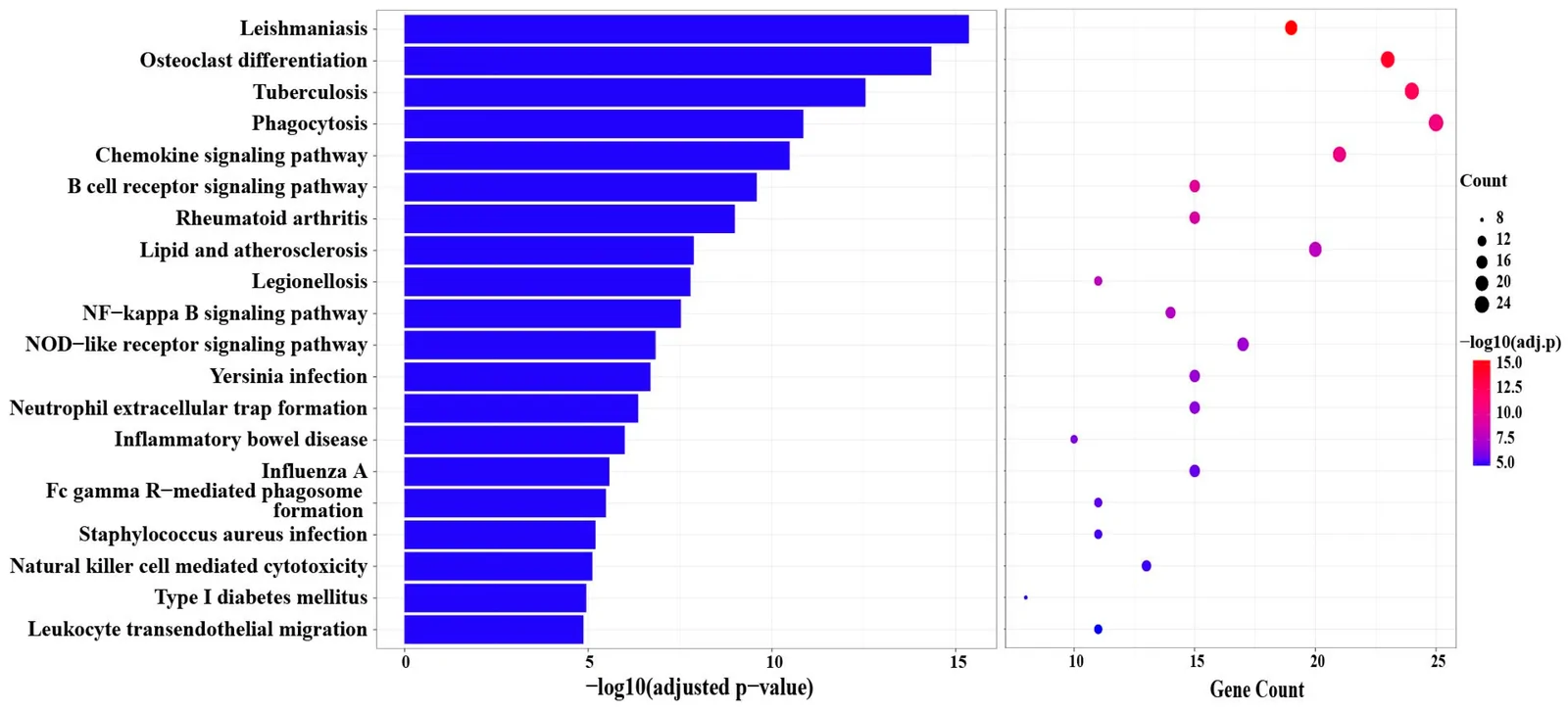
KEGG pathway enrichment analysis of genes affected by *RHOA* virtual knockout.

KEGG enrichment analysis demonstrated that *RHOA* perturbation significantly affected multiple immune- and inflammation-related pathways (Figure 14; Supplementary Table S18). The enriched pathways included Leishmaniasis, Osteoclast differentiation, Tuberculosis, Phagocytosis, Chemokine signaling pathway, B cell receptor signaling pathway, Rheumatoid arthritis, Lipid and atherosclerosis, and NF-kappa B signaling pathway.

**Figure 15:**
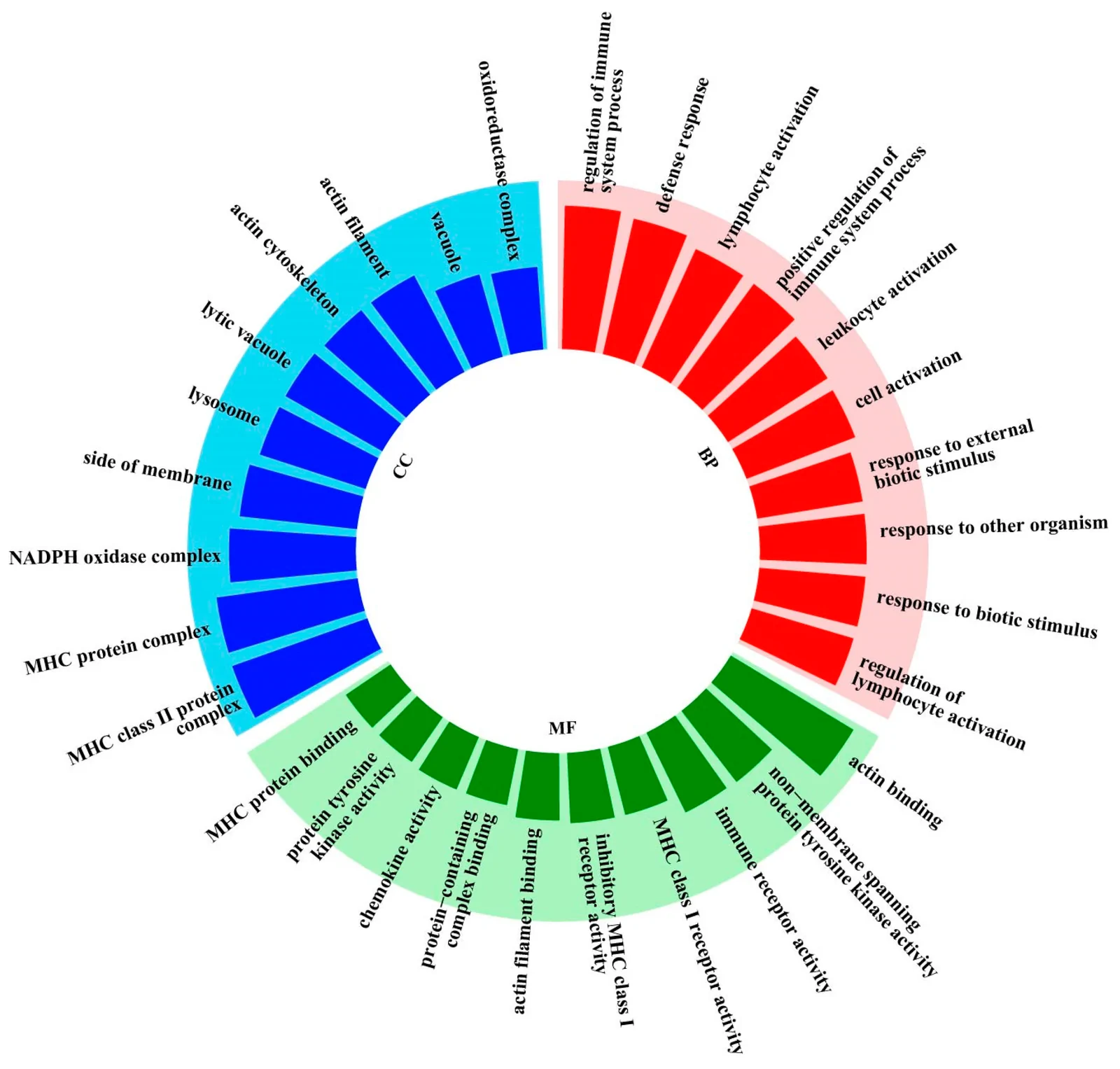
GO enrichment analysis of genes affected by *RHOA* virtual knockout. Red, blue, and green sectors represent biological process (BP), cellular component (CC), and molecular function (MF) categories, respectively.

GO enrichment analysis further revealed that *RHOA* perturbation was associated with immune-related transcriptional programs within the integrated bovine lung cell population. (Figure 15; Supplementary Tables S19–S21). At the BP level, affected genes were enriched in immune system process regulation, defense response, leukocyte activation, and response to biotic stimulus. CC enrichment highlighted membrane side, lysosome, NADPH oxidase complex, and MHC protein complex, whereas MF categories were mainly associated with protein binding, actin binding, kinase activity, and MHC protein binding.

**Figure 16:**
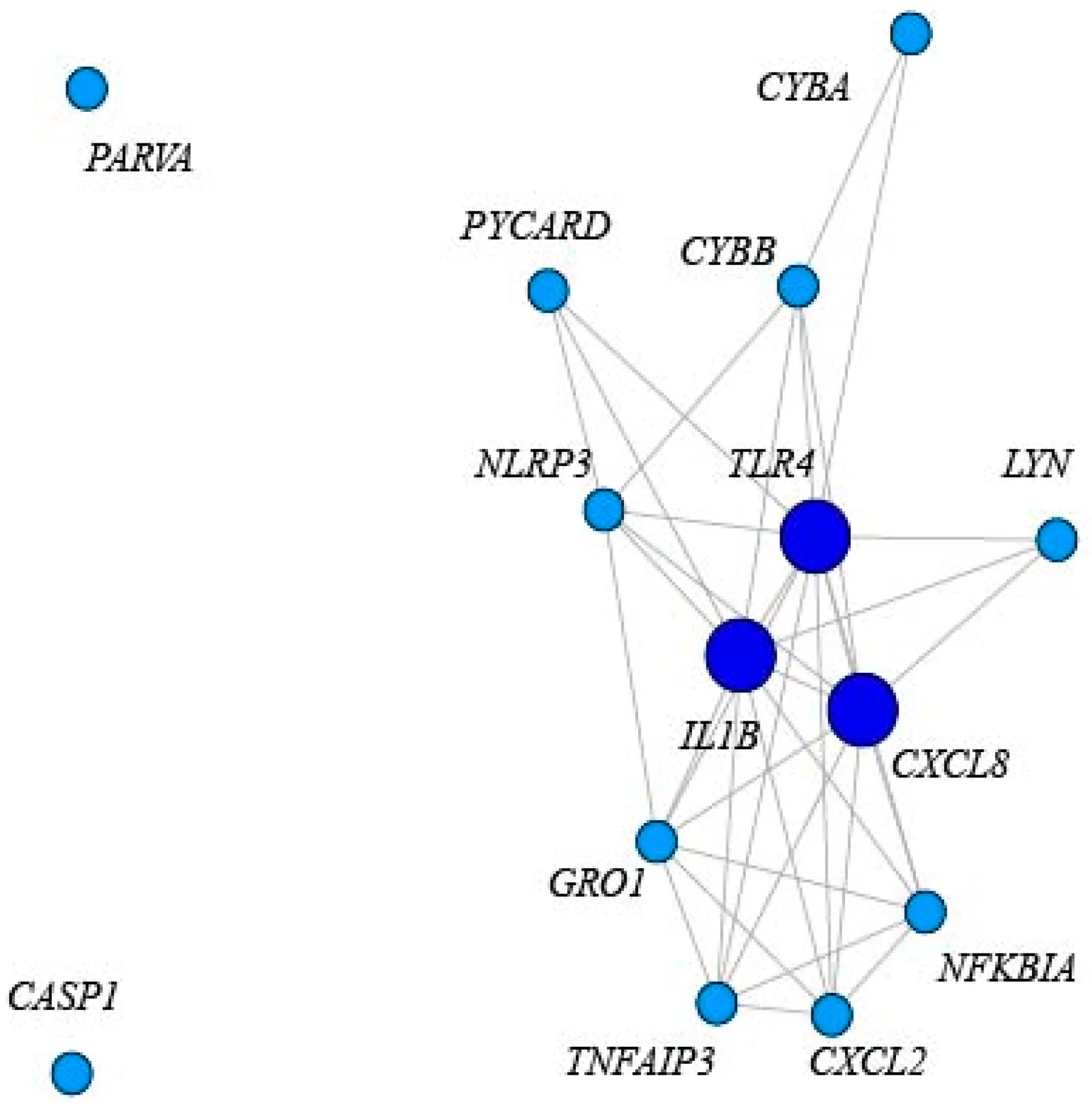
Protein–protein interaction network of genes affected by *RHOA* virtual knockout. Node size and color intensity represent node degree, with larger and darker nodes indicating higher connectivity; gray edges represent protein–protein interactions. Blue-highlighted gene la-bels indicate hub genes.

PPI network analysis identified a tightly connected network containing 14 nodes and 37 edges (combined score ≥ 400) among *RHOA*-associated differentially regulated genes (Figure 16). Network analysis revealed that *TLR4*, *IL1B*, *CXCL8*, *CYBB*, *CYBA*, *NLRP3*, *PYCARD*, *LCP1*, *TYROBP* and *FCER1G* occupied central positions within the interaction network, exhibiting high connectivity and suggesting their potential roles as key regulatory nodes in immune-inflammatory signaling.

## 4. Discussion

In this study, we leveraged publicly available whole-genome resequencing data from 70 Ethiopian indigenous cattle individuals to characterize their population genomic landscape and investigate the genetic basis underlying high-altitude adaptation in livestock from the Horn of Africa. By integrating genome-wide selective sweep analyses with single-cell transcriptome-based in silico virtual knockout approaches, we identified candidate adaptive mechanisms involving cardiovascular homeostasis, ECM remodeling, and immune balance. Our findings reveal that Ethiopian indigenous cattle have evolved a distinct genomic strategy to cope with extreme high-altitude environments, involving coordinated regulation across multiple physiological systems.

Population genetic analyses revealed pronounced differentiation between low-altitude cattle populations (for example, Abigar) and mid-to-high-altitude populations (including Barka, Felata, Fogera, and Horro). Both PCA and phylogenetic reconstruction supported clear genetic separation between these groups. In addition, linkage disequilibrium (LD) decay analyses revealed differences in genomic linkage patterns among Ethiopian indigenous cattle populations. The overall rapid decline of LD with increasing physical distance indicated extensive historical recombination across populations, whereas differences in LD persistence suggested variation in demographic history and genome-wide linkage structures. Nevertheless, several limitations should be acknowledged. Altitude-associated genomic differences in this study were inferred from comparisons among Ethiopian indigenous cattle breeds distributed across different elevations. Because breed identity, geographic origin, and altitude are correlated in the current sampling design, some selective signals may also reflect breed history, geographic isolation, demographic history, or artificial selection. To evaluate whether population structure inference was affected by linkage disequilibrium or cryptic relatedness, we additionally performed PCA and ADMIXTURE analyses based on LD-pruned SNP datasets and relatedness-controlled subsets. These analyses showed that the major clustering patterns were largely consistent after controlling for these potential biases; however, breed-associated clustering and partial overlap among altitude categories remained evident. Therefore, the observed genomic differentiation likely reflects a combination of breed background, demographic history, geographic differentiation, and environmental pressures rather than altitude alone. Accordingly, the identified loci should be considered candidate altitude-associated genomic signatures or potential adaptive signals rather than definitive altitude-specific causal variants. Future studies incorporating larger sample sizes, geographically replicated populations, and genetically matched populations across comparable altitude gradients will be required to more rigorously distinguish altitude-associated selection from lineage-specific evolutionary effects. These populations exhibit distinct genomic backgrounds that may reflect the combined effects of demographic history, geographic differentiation, breed history, and environmental selection pressures. The observed differentiation in population structure together with variation in LD decay patterns suggests that the genomic architectures of Ethiopian indigenous cattle have been shaped by the combined effects of demographic history, geographic separation, and environmental pressures. Populations with slower LD decay may have experienced reduced effective population size, historical isolation, or stronger retention of ancestral linkage blocks, whereas faster LD decay may indicate more extensive historical recombination and greater genomic reshuffling. However, these demographic interpretations should be considered cautiously because LD patterns can be influenced by multiple interacting factors, including population history, sampling structure, and historical gene flow. Similar altitude-associated genetic differentiation has also been reported in Tibetan cattle and yak populations inhabiting the Qinghai–Tibetan Plateau [45,46]. Low-altitude populations may retain adaptive characteristics associated with tropical and subtropical environments, potentially including heat tolerance and parasite resistance. In contrast, although mid-to-high-altitude breeds formed a distinct lineage, their individuals exhibited considerable genetic admixture, indicating ongoing gene flow among highland populations. Such gene flow may have resulted from historical migration, livestock exchange, or seasonal pastoral movements, facilitating the dissemination of adaptive alleles across highland populations and potentially accelerating collective adaptation to similar environmental pressures. ADMIXTURE analysis further supported the shared ancestry and genetic similarity among high-altitude populations, suggesting that these breeds share partially overlapping genetic backgrounds, although the contribution of historical gene flow and environmental selection cannot be completely disentangled, particularly for environmental pressures such as hypoxia and cold stress. Similar patterns of adaptive introgression have been observed between yak and Tibetan cattle, where gene exchange contributed to environmental adaptation [47]. This combination of strong differentiation between ecological zones and extensive connectivity among highland populations may preserve genetic diversity while maintaining evolutionary potential under fluctuating environmental conditions.

Genome-wide selective sweep analyses identified 398 candidate genomic regions showing concordant signals in both the *F_ST_* and θπ scans. These regions represent overlaps between two complementary selection-scan metrics rather than genomic regions necessarily shared across all cattle populations. Because *F_ST_* captures population differentiation whereas θπ reflects differences in nucleotide diversity, the concordance between these two metrics provides stronger support for these regions as candidates affected by selection. Functional enrichment analysis revealed significant enrichment of five major signaling pathways, including phospholipase D, Ras, ErbB, Wnt, and Hedgehog signaling pathways. These pathways are involved in vascular regulation, cellular survival, energy metabolism, and tissue remodeling, and show substantial overlap with adaptive pathways previously reported in Tibetan Plateau species [14,48,49]. The phospholipase D pathway contributes to membrane lipid metabolism and cytoskeletal organization, potentially maintaining cellular integrity under hypoxic and cold stress. The Ras signaling pathway acts as an upstream regulatory hub integrating growth factor and stress signals and modulating downstream PI3K-Akt and MAPK pathways involved in cell survival, proliferation, and apoptosis regulation [50]. The ErbB signaling pathway has been implicated in cardiopulmonary remodeling, and its activity is essential for maintaining pulmonary vascular function and preventing excessive hypoxia-induced vascular responses [51]. Structural variation affecting EGFR, a member of the ErbB family, in yak genomes further supports the evolutionary plasticity of this pathway during high-altitude adaptation [52]. The Wnt pathway regulates skeletal development, lipid metabolism, and cell fate decisions under hypoxic conditions. Through interactions with HIF-1α, Wnt signaling can promote VEGF expression and angiogenic responses [53,54]. In addition, its interaction with PI3K-Akt signaling contributes to metabolic adaptation under oxygen limitation [55]. Similar PI3K-related regulatory patterns have also been identified among candidate adaptive genes in Tibetan sheep [49]. Meanwhile, Hedgehog signaling participates in organ development, angiogenesis, and metabolic regulation, suggesting that these conserved developmental pathways may contribute to adaptive remodeling under chronic hypoxia. Among the candidate genes identified in this study, *PIK3CA*, encoding the catalytic subunit of phosphoinositide 3-kinase (PI3K), represents a potential central regulator connecting vascular adaptation, metabolism, and tissue remodeling. The PI3K-Akt pathway promotes HIF-1α activation and VEGF expression, while simultaneously regulating glucose metabolism and cellular energy reprogramming [56]. Under hypoxic conditions, PI3K-Akt signaling facilitates the metabolic transition from oxidative phosphorylation toward glycolysis, thereby matching energy production with reduced oxygen availability. Similar mechanisms have been reported in Tibetan pigs and Wutuo black sheep [57]. In addition, PI3K-Akt signaling protects vascular endothelial and cardiac cells by suppressing mitochondrial apoptosis and enhancing antioxidant responses [58,59]. Positive selection signals within this pathway have been repeatedly identified in high-altitude-adapted animals [60,61]. Although the integration of FST and θπ signals provides complementary evidence for prioritizing candidate differentiated genomic regions, this approach remains an empirical outlier-based framework. The overlap between the two statistics increases confidence in candidate regions but does not independently prove positive selection or establish altitude as the exclusive selective force. Therefore, these regions should be interpreted as candidate genomic regions associated with environmental differentiation, requiring further validation using additional population genetic approaches, replicated sampling designs, and functional experiments.

Given the well-established role of *PIK3CA* in PI3K–Akt signaling and hypoxia-related metabolic regulation, we next explored its potential downstream regulatory consequences using in silico virtual knockout analysis [56,59,61]. Our single-cell transcriptome-based virtual knockout analysis further revealed a potential role of *PIK3CA* in extracellular matrix (ECM) remodeling during hypoxic adaptation. Following simulated *PIK3CA* disruption, differentially regulated genes were significantly enriched in pathways associated with ECM organization, focal adhesion, and integrin signaling, with key hub genes including *COL1A1*, *COL1A2*, *FN1*, *LAMA4*, and *FBN1*. Consistently, KEGG enrichment analysis indicated significant perturbation of the ECM–receptor interaction, Focal adhesion, and TGF-beta signaling pathways [62,63]. The ECM not only provides structural support for the alveolar–capillary barrier but also regulates endothelial cell survival and permeability through integrin-mediated mechanotransduction [64,65]. Under hypoxic conditions, *PIK3CA* may regulate collagen synthesis through activation of downstream PI3K–Akt targets, including phosphorylation of GSK-3β and β-catenin, thereby modulating the TGF-β/Smad signaling axis [66,67]. In addition, *PIK3CA* may influence the balance between ECM degradation and deposition through Rac1-dependent cytoskeletal remodeling. Collectively, these findings suggest that *PIK3CA* may be associated with extracellular matrix-related transcriptional responses during hypoxic adaptation, potentially linking metabolic regulation with tissue remodeling [68]. Therefore, precise regulation of *PIK3CA* activity may represent a critical determinant balancing adaptive remodeling and pathological tissue fibrosis under chronic hypoxic stress.

*RHOA* regulates vascular smooth muscle contraction and remodeling through the RHOA/ROCK signaling pathway, which has been established as a critical regulator of vascular tone and pulmonary vascular remodeling, particularly during the development of hypoxia-induced pulmonary arterial hypertension (HPAH) [50]. The importance of this pathway in hypoxic adaptation appears to be evolutionarily conserved across species. In humans, excessive activation of RHOA/ROCK signaling contributes to pathological vascular alterations associated with high-altitude disorders [69,70] whereas in naturally adapted species, appropriate modulation of this pathway may facilitate vascular homeostasis under chronic hypoxic stress. Following virtual knockout of *RHOA*, differentially regulated genes were significantly enriched in immune- and inflammation-related pathways, including NF-κB signaling, phagocytosis, chemokine signaling, and B cell receptor signaling pathways. Core immune-inflammatory regulators, including *TYROBP*, *FCER1G*, *IL1B*, *TLR4*, and *NLRP3*, exhibited strong transcriptional activation following *RHOA* perturbation [71,72]. GO enrichment analysis further demonstrated enrichment of immune-related processes, including leukocyte activation, defense response, and regulation of immune system processes. These findings suggest that *RHOA* may participate in both vascular regulation and immune-related transcriptional responses during environmental stress adaptation [73,74]. In addition, Chronic hypoxia can induce sterile inflammation through ROS accumulation and HIF-1α-dependent mechanisms, leading to NF-κB activation and NLRP3 inflammasome assembly [75,76]. Notably, livestock populations in the Horn of Africa are frequently exposed to tropical pathogens, including trypanosomes and Babesia parasites., which may impose additional immune challenges [77,78]. In the present study, *RHOA* was identified as a candidate gene associated with high-altitude adaptation in Ethiopian cattle. Its potential functional relevance is consistent with previous findings involving vascular regulatory genes such as *EGFR* in yak [52] and the influence of *Epas1* genotypes on cardiovascular function in deer mice [79,80]. Together, these findings highlight the potential role of *RHOA*-mediated regulation of vascular and immune homeostasis in coping with chronic hypoxic environments.

Our results suggest that *PIK3CA* and *RHOA* may represent two candidate genes associated with distinct biological processes related to high-altitude adaptation. PI3K-Akt signaling can influence *RHOA* activation through lipid-mediated recruitment of guanine nucleotide exchange factors, whereas RHOA/ROCK signaling can regulate Akt activity through feedback involving PTEN and LIM kinase pathways [81,82]. Such reciprocal regulation may prevent excessive vascular remodeling while maintaining structural flexibility during long-term hypoxic exposure. Our in silico perturbation results further support complementary functions of these two regulators: *PIK3CA* disruption primarily affected endothelial and basement membrane-associated structural programs, whereas *RHOA* disruption enhanced immune-inflammatory responses. Interestingly, the adaptive genetic architecture identified in Ethiopian cattle differs from the classical *EPAS1*-centered model widely reported in Tibetan and Andean high-altitude populations. *EPAS1* has repeatedly shown strong signatures of positive selection across multiple Tibetan livestock species [46,83,84,85,86] and Andean livestock populations also exhibit enrichment of HIF pathway genes, including *EPAS1* and *EGLN1* [87,88,89]. However, *EPAS1* was not identified as a significant selective candidate in Ethiopian cattle in this study, consistent with previous analyses of Ethiopian livestock species, including cattle, sheep, and goats [29,90,91,92]. This difference may reflect distinct evolutionary trajectories shaped by geographic history, domestication processes, genetic background, and local environmental pressures. Our findings therefore suggest that high-altitude adaptation may involve lineage-specific molecular strategies associated with regional ecological challenges, although the contribution of altitude itself cannot be completely separated from breed history and demographic processes in the current dataset. Such lineage-specific strategies may have arisen from differences in demographic history, domestication, genetic background, and local selective pressures, which could have favored alternative molecular routes for adaptation to chronic hypoxia [93]. The virtual knockout analysis was based on Bos taurus lung single-cell transcriptomic data rather than Ethiopian highland cattle-specific datasets. Thus, the predicted regulatory effects of *PIK3CA* and *RHOA* should be interpreted as conserved bovine regulatory responses rather than direct representations of Ethiopian cattle-specific networks. Future generation of highland cattle single-cell datasets will be important for validating these regulatory relationships. Because the virtual knockout analysis was performed using an integrated lung cell population, some predicted regulatory responses may reflect cell-type-specific expression architectures; future cell-type-resolved perturbation analyses will be required. Although our genomic and computational analyses provide evidence supporting potential roles of *PIK3CA* and *RHOA* in altitude-associated adaptive processes, these findings remain hypothesis-generating. Further functional validation using hypoxia-exposed bovine pulmonary cells, gene perturbation approaches (e.g., CRISPR/Cas9 or RNA interference), and integrated transcriptomic and physiological analyses will be required to confirm their contributions to adaptive variation in Ethiopian cattle.

## 5. Conclusions

In this study, we integrated whole-genome resequencing and single-cell transcriptome-based in silico virtual knockout analyses to systematically investigate the genomic basis underlying high-altitude adaptation in Ethiopian indigenous cattle. Population genomic analyses revealed pronounced genetic differentiation between low-altitude and mid-to-high-altitude populations, whereas extensive gene flow was observed among high-altitude populations, suggesting potential routes for the dissemination of adaptive alleles within elevated environments. Genome-wide selective sweep analyses identified 398 shared candidate genomic regions encompassing 1816 genes, which were significantly enriched in hypoxia-related and vascular regulatory pathways, including Phospholipase D, Ras, ErbB, Wnt, and Hedgehog signaling pathways. Notably, in silico perturbation analyses predicted distinct regulatory effects associated with *PIK3CA* and *RHOA*. PIK3CA perturbation was associated with changes in extracellular matrix organization, focal adhesion, and integrin signaling networks, suggesting its potential involvement in pulmonary structural regulation under hypoxic conditions. In contrast, *RHOA* perturbation affected immune-inflammatory pathways, including NF-κB/NLRP3-associated responses, suggesting its potential contribution to immune regulation during chronic environmental stress.

Together, these findings suggest that *PIK3CA* and *RHOA* associated regulatory networks may represent candidate components linking tissue structural maintenance and immune regulation during high-altitude adaptation. This study provides genomic and transcriptomic evidence for previously underexplored adaptive mechanisms in African livestock and expands our understanding of lineage-specific strategies underlying mammalian adaptation to extreme environments. Furthermore, the identified candidate genes and pathways provide potential molecular targets for genetic improvement and conservation of high-altitude cattle resources.

## Figures and Tables

**Table 1:** Ethiopian cattle breeds and corresponding sampling locations.

Breeds	No. of Samples	Geographic Region	Altitude (m)	Latitude	Longitude	Agro-Ecology
Abigar	10	Gambela	523	8.123469	34.30687	Hot, humid, and low-altitude
Barka	10	Amhara	895	14.18467	36.89087	Hot, humid, and low-altitude
Boran	10	Oromiya	1368	4.978936	38.27516	Hot, humid, and mid-altitude
Felata	10	Amhara	552	12.40733	35.87573	Hot, humid, and low-altitude
Fogera	10	Amhara	1735	11.86045	37.81373	Humid and mid altitude
Gojjam-Highland	10	Amhara	3410	10.72113	37.85988	Cold, humid, and high-altitude
Horro	10	Oromiya	1722	9.672949	37.07545	Humid and mid-altitude

## Data Availability

The raw whole-genome resequencing data used in this study were obtained from the NCBI Sequence Read Archive (SRA) under accession numbers SRP478348 and SRP480803. These datasets were originally produced by Ayalew et al. and are publicly available. The single-cell RNA sequencing data used for in silico virtual knockout analyses were retrieved from the NCBI Gene Expression Omnibus (GEO) database under accession number GSE266061, which includes lung single-cell transcriptomic profiles of yak, cattle–yak hybrids, and cattle. All datasets are publicly accessible and were used in accordance with the data access policies of the respective repositories.

## Supplementary Materials

The following supporting information can be downloaded at: https://www.mdpi.com/article/10.3390/biology15201822/s1, Table S1: Sample description; Table S2: Sequencing depth of the 70 Ethiopian indigenous cattle samples; Table S3: Chromosome-level nucleotide diversity; Table S4: Main functional categories of identified SNPs; Table S5: Detailed functional annotation of identified SNPs; Table S6: Chromosome-level SNP density; Table S7: Genome-wide windowed Weir and Cockerham FST estimates; Table S8: Windowed nucleotide diversity (π) estimates for low-altitude cattle populations; Table S9: Windowed nucleotide diversity (π) estimates for mid-to-high-altitude cattle populations; Table S10: Integrated window-based FST and nucleotide diversity statistics; Table S11: KEGG pathway enrichment results for genes in candidate selective regions; Table S12: Differentially regulated genes following PIK3CA virtual knockout; Table S13: KEGG enrichment results for genes affected by PIK3CA virtual knockout; Table S14: GO biological process enrichment results for genes affected by PIK3CA virtual knockout; Table S15: GO cellular component enrichment results for genes affected by PIK3CA virtual knockout; Table S16: GO molecular function enrichment results for genes affected by PIK3CA virtual knockout; Table S17: Differentially regulated genes following RHOA virtual knockout; Table S18: KEGG enrichment results for genes affected by RHOA virtual knockout; Table S19: GO biological process enrichment results for genes affected by RHOA virtual knockout; Table S20: GO cellular component enrichment results for genes affected by RHOA virtual knockout; Table S21: GO molecular function enrichment results for genes affected by RHOA virtual knockout; Figure S1: Relatedness-controlled principal component analysis (PCA) of Ethiopian indigenous cattle; Figure S2: ADMIXTURE ancestry profiles of 70 Ethiopian indigenous cattle from K = 2 to K = 7; Figure S3: Relatedness-controlled ADMIXTURE ancestry profiles of 57 Ethiopian indigenous cattle from K = 2 to K = 7; Figure S4: Flowchart of the identification of 398 candidate selective regions.
