# Supplementary material for: Whole-Genome Resequencing Reveals Genomic Signatures Underlying High-Altitude Adaptation in Ethiopian Indigenous Cattle
Source: Biology (Basel). 2026 Oct 12;15(20):1822. doi: 10.3390/biology15201822 (PMC13701653; doi:10.3390/biology15201822)
Supplement: Supplementary file 1 [file biology-15-01822-s001.zip › supplementary_Figure.pdf]

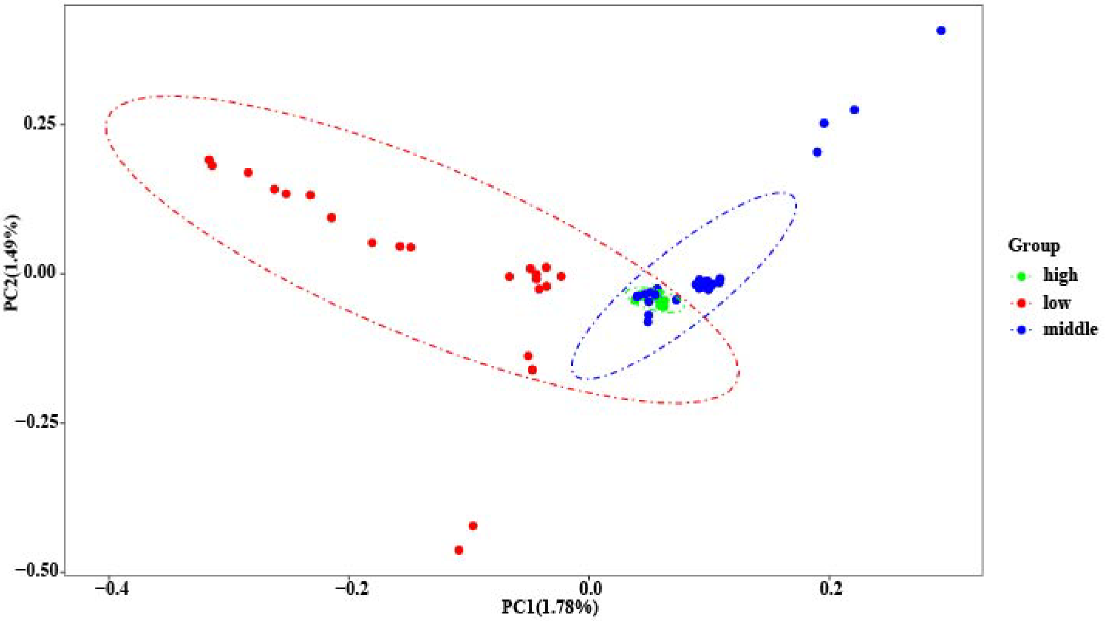

Supplementary Figure S1. Relatedness-controlled principal component analysis (PCA) of Ethiopian indigenous cattle. PCA was performed using 1,435,022 LD-pruned autosomal SNPs after excluding individuals involved in second-degree or closer relationships based on a KING kinship coefficient cutoff of 0.0884. A total of 57 relatively unrelated individuals were retained for the sensitivity analysis. Individuals are colored according to low-, middle-, and high-altitude categories.

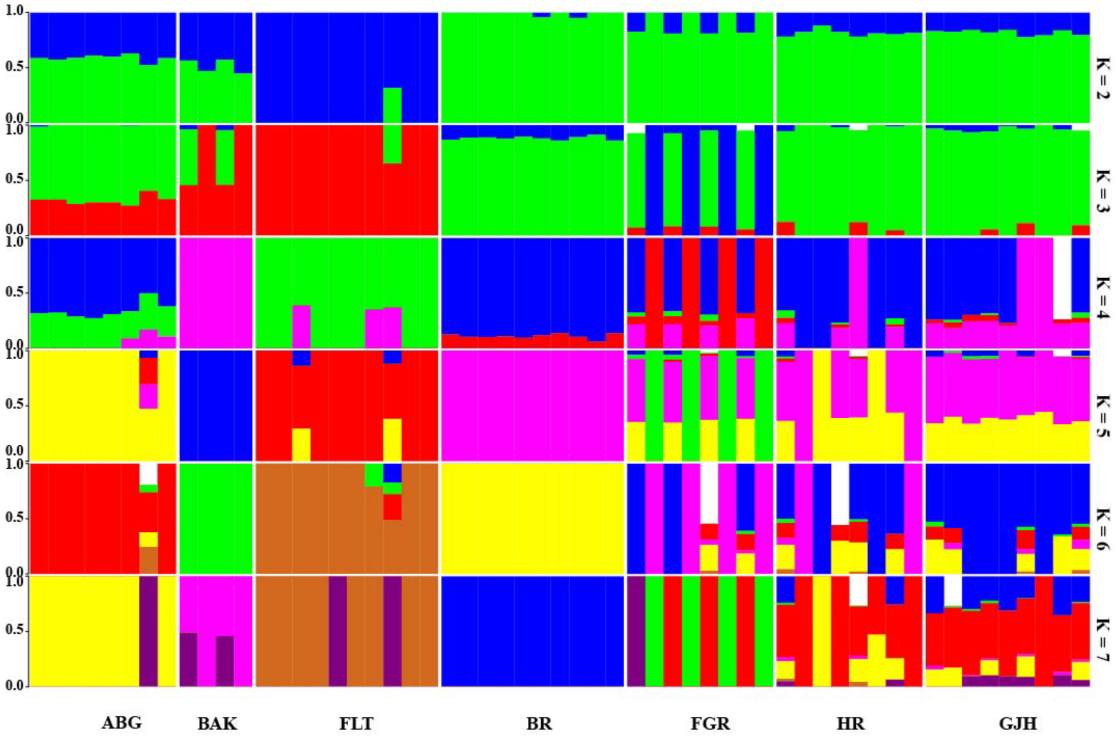

Supplementary Figure S2. ADMIXTURE ancestry profiles of 70 Ethiopian indigenous cattle from K = 2 to K = 7. ADMIXTURE analysis was performed using 1,435,022 LD-pruned autosomal SNPs. Individuals are arranged according to the seven cattle populations (ABG, BAK, FLT, BR, FGR, HR, and GJH), which are further grouped into low-, middle-, and

high-altitude categories. Increasing K values illustrate progressively finer patterns of population- and breed-associated ancestry composition.

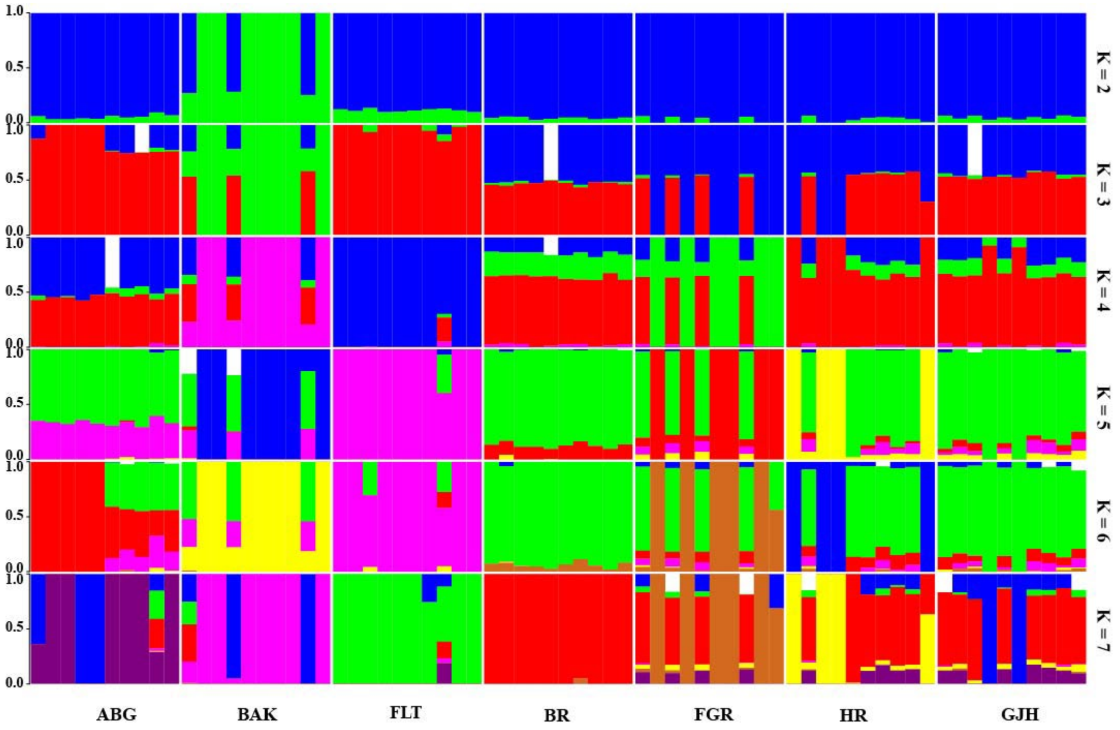

Supplementary Figure S3. Relatedness-controlled ADMIXTURE ancestry profiles of 57 Ethiopian indigenous cattle from K = 2 to K = 7. Individuals involved in second-degree or closer relationships were excluded using a KING kinship coefficient cutoff of 0.0884, leaving 57 relatively unrelated individuals. ADMIXTURE analysis was performed using the same set of 1,435,022 LD-pruned autosomal SNPs. Individuals are arranged according to the seven cattle populations and their corresponding low-, middle-, and high-altitude categories.

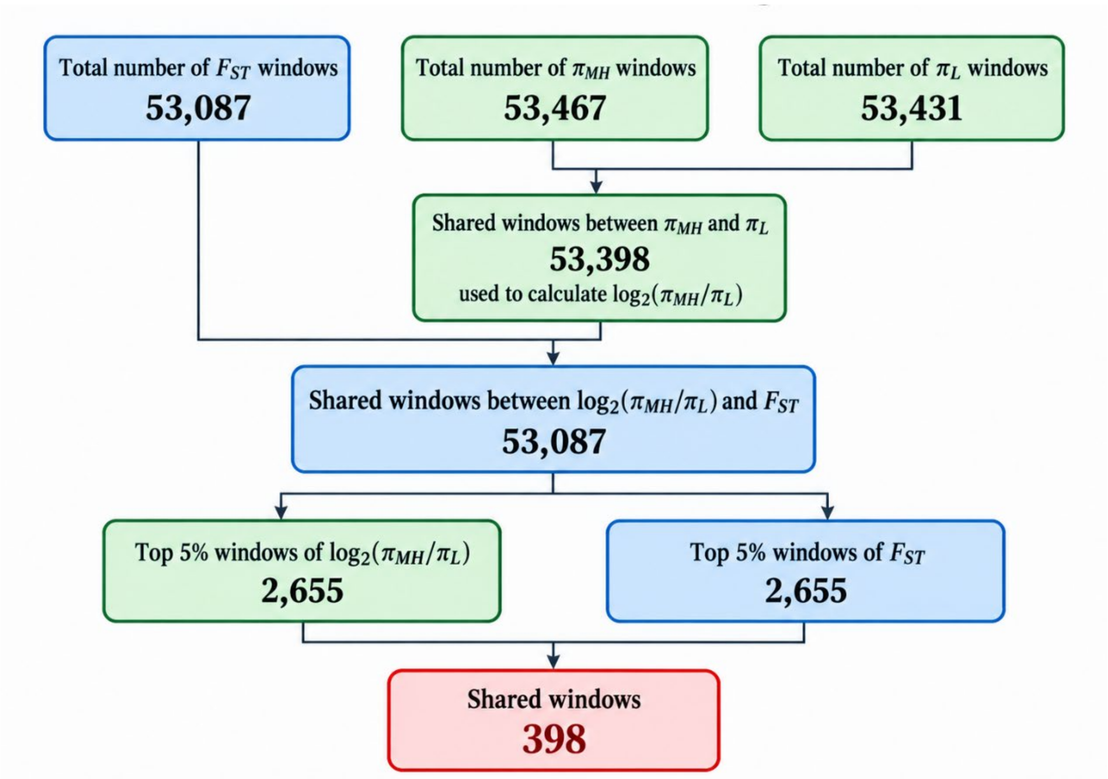

Supplementary Figure S4. Flowchart of the identification of 398 candidate selective regions.
